# Improving gene expression data interpretation by finding latent factors that co-regulate gene modules with clinical factors

**DOI:** 10.1186/1471-2164-12-563

**Published:** 2011-11-16

**Authors:** Tianwei Yu, Yun Bai

**Affiliations:** 1 Department of Biostatistics and Bioinformatics, Rollins School of Public Health, Emory University, Atlanta, GA, USA; 2 Department of Pharmaceutical Sciences, School of Pharmacy, Philadelphia College of Osteopathic Medicine, Suwanee, GA, USA

## Abstract

**Background:**

In the analysis of high-throughput data with a clinical outcome, researchers mostly focus on genes/proteins that show first-order relations with the clinical outcome. While this approach yields biomarkers and biological mechanisms that are easily interpretable, it may miss information that is important to the understanding of disease mechanism and/or treatment response. Here we test the hypothesis that unobserved factors can be mobilized by the living system to coordinate the response to the clinical factors.

**Results:**

We developed a computational method named Guided Latent Factor Discovery (GLFD) to identify hidden factors that act in combination with the observed clinical factors to control gene modules. In simulation studies, the method recovered masked factors effectively. Using real microarray data, we demonstrate that the method identifies latent factors that are biologically relevant, and extracts more information than analyzing only the first-order response to the clinical outcome.

**Conclusions:**

Finding latent factors using GLFD brings extra insight into the mechanisms of the disease/drug response. The R code of the method is available at http://userwww.service.emory.edu/~tyu8/GLFD.

## Background

When high-throughput biomedical data are collected together with outcome variables, such as treatment groups or drug response, the focus of data analysis is mainly selecting features that correlate with the outcome variables and building predictive models [1,2]. Analysis at the functional group (gene set) level is also popular because it provides mechanistic understanding and helps reduce the search space for feature selection [3-5]. Such methods mostly focus on finding gene sets that show first-order relationship with the clinical outcome variable.

The biological system is a complex network, and even genes involved in the same biological process may not be correlated [6-8]. Rather, more complex relations such as dynamic correlation exist [5,9,10]. Thus we expect the response to the clinical variable is not limited to first-order relations, and more complex molecular events are involved. For mechanistic studies, it may be important to find molecular events that occur in association with the clinical outcome, but are not correlated with the clinical outcome in first order.

We try to address this issue using the latent factor model approach, which has been successful in modeling gene regulatory networks [11]. It has been established that the complex biological system is of modular structures [12,13], and the gene expression within a module can be modeled reasonably well by linear functions of the activities of the controlling factors [11,14,15]. When using latent factor models, in some situations the latent factors carry physical meaning, such as transcription factors (TF) in gene expression [14]. In other situations, the latent factors may be combinations of true biological factors, or simply some virtual controllers that reflect the collective behavior of groups of genes/proteins [15]. Thus we do not imply causal relationships by using the factor model, and the word "regulate" is used in a loose manner in this manuscript.

In the situation where observable clinical factors are exerted on the system, we hypothesize that the biological system would mobilize other unobserved factors to coordinate the response to the clinical factors, while the response is limited to certain relevant modules. In this manuscript, we test this hypothesis by developing a new method named Guided Latent Factor Discovery (GLFD) to find such factors if they exist. The method is based on the modular decomposition of large matrices [15]. By analyzing real datasets, we demonstrate that such latent factors do exist, and they bring extra insight into the interpretation of the data. The R code of the method is available at http://userwww.service.emory.edu/~tyu8/GLFD.

## Methods

### The model

Consider a data matrix ***G***_*p × n *_with *p *genes measured in *n *samples, and let ***B***_*n × m *_be the matrix of the scores of *m *known clinical factors, e.g. treatment groups or measured responses. Our goal is to search for a group of hidden factors, ***F***_*n × r*_, such that ***B ***and ***F ***jointly regulate a gene module, with relationships represented by a linear factor model,

(1) $$G_{q\times n}^{\left(module\right)}=L_{q\times \left(m+r\right)}{\left[B_{n\times m}F_{n\times r}\right]}^{T}+E_{q\times n}$$

where *q *is the number of genes in the module, ***L ***is the regulation strength matrix, and ***E ***is the residual matrix. The number of genes, *q*, is usually much less than the number of genes in the data matrix *(p)*, as only a fraction of genes are expected to be regulated by the clinical factor and the latent factors. To qualify as a module, a significant portion of the selected genes need to have non-zero loadings on both ***B ***and ***F***.

### The procedure to find latent factors

We develop a three-step procedure to find the latent factors.

#### Step 1. Finding weighted residual from each gene

In this step the residual of every gene is taken after projecting on the clinical factors. The residuals are then weighted based on the level of association between the gene and the clinical factors.

(a) Standardize the gene expression vectors such that each row-vector of ***G ***is unit length. Standardize the clinical factor matrix such that the column vectors of ***B ***are unit length and orthogonal to each other. This is done by using the whitening transformation. Briefly, let ***Λ ***be the diagonal matrix of eigenvalues of ***B***^***T***^***B***, and ***Φ ***be the corresponding matrix of eigen vectors as its columns, then we take B* = BΦΛ^***-1/2***^. The column vectors of B* form an orthonormal basis and span the same subspace.

(b) Project each row vector of ***G***, ***g***_***i ***_onto B*, and find the projection length,

(2) $$l_{i}=\sqrt{g_{i}B^{*}B^{{*}^{\prime }}{g_{i}}^{\prime }}$$

(c) Take the residual of each gene after projection onto the clinical factors. Let ***β***_1_, ..., ***β***_*m *_be the column vectors of B* ,

(3) $$r_{i}=g_{i}-\sum_{j=1}^{m}\left(g_{i}\beta_{j}\right)\beta_{j}^{\prime }$$

(d) This step is to assign weight to each residual such that contribution to subspace finding is mostly limited to genes significantly associated with the clinical outcome. Weigh each residual vector based on the gene's projection length using a sigmoid function:

(4) $$r_{i}^{*}=\left(1-\frac{1}{1+e^{\varphi \left(l_{i}-\delta \right)}}\right)r_{i}$$

where φ is a large value, e.g. 100, to make the sigmoid function approach a step function. When φ is large enough, further increasing its value has little impact on the shape of the curve. The inflection point of the sigmoid curve, δ, is determined by the probability of the gene being independent from ***B***. It is based on the fact that the projection length of a gene independent of the factors follows the *F *distribution [16].

(5) $$\delta =\sqrt{\frac{mF_{1-\alpha ,m,n-m-1}}{\left(n-m-1\right)+mF_{1-\alpha ,m,n-m-1}}}$$

where *n *is the number of samples, and *m *is the number of factors in ***B***. A stringent α level cutoff, e.g. 0.001 is used to account for the multiplicity caused by the large number of genes under study. This value yields an expected one false positive for every 1000 features. The choice is dependent on the number of features being studied. A more stringent cutoff needs to be used when a higher number of features are involved. Following *eq.5*, the value of *δ *is equal to the projection length that corresponds to the alpha level. Residuals of genes with projection length higher than *δ *receive weights close to 1, while those lower than *δ *receive weights close to zero.

#### Step 2. Searching for modules in the weighted residual matrix

This part of the procedure is based on our method Modular Latent Structure Analysis (MLSA) [15], which searches for gene modules regulated by linear combinations of latent factors. Briefly, MLSA seeks subspaces on which a portion of the row-vectors have large projection length. It assumes no prior knowledge about module membership. The logic behind the method is that row-vectors belonging to a module controlled by some latent factors should have big projection lengths on the subspace spanned by those latent factors. A module is defined as a group of row-vectors whose values are controlled by the same set of latent factors. Combinatorial effects between the latent factors are necessary for the factors to belong to the same module [15].

When the dimensionality of the subspace is known, MLSA uses an EM-like algorithm iterating between (a) reweighting each row-vector based on its association with the current factor estimates, and (b) re-estimating the latent factors of the module, until convergence. In most cases the dimensionality is unknown, in which case MLSA uses step forward search to determine the dimensionality of a module. Multiple modules can be identified from a dataset.

(a) We take the weighted residual matrix ***R ***from Step 1, each row vector of which is the weighted residual of a gene. We first find the length of the longest weighted residual vector, $l_{max}=max_{i}||r_{i}^{*}||$, where $r_{i}^{*}$ is the *i*^*th *^row vector. We then divide the matrix by this value,

(6) $$R^{*}=R∕l_{max}$$

This step makes the maximum row-vector length one. It replaces the data standardization step of MLSA, which standardizes every row vector to length one. MLSA makes inference based on projection length. This new procedure makes sure that contributions to latent factor finding come mostly from genes significantly associated with the clinical factor set ***B***.

(b) We then iteratively find modules from R*.

(b.1) With each of the dimensionality values *k *= *l*, ..., *K*, use the EM-like algorithm to find a module from the data matrix (Algorithm 1 in [15]). The maximum allowable dimensionality value *K *is taken such that no module is likely to exceed this value. In the current study we used *K *= 10, which means the maximum allowable dimensionality of a module is 10 dimensions. For every *k*, instead of randomly initiating the latent factor estimates, we start the search from the first *k *right singular vectors of the data matrix. Thus the algorithm is less likely to converge to a local optimum.

(b.2) Compare the sizes of the modules across different dimensionality *(k)*, and select the module that contains the largest number of genes. The number of associated genes is determined by an inference procedure that depends on the dimensionality of the subspace, i.e. longer projection length is required for a higher dimensional subspace [15].

(b.3) If the number of genes in the newly found module is less than a small threshold, e.g. 10 genes, we end the iteration. Else, for every row vector of the matrix, we subtract its projection onto the basis of the module. Using the new residual matrix, return to step (b.1) to find another module.

The result from this step is a collection of latent factor sets (module basis), i.e. matrices with latent factor scores in the column, $\left\{F_{n\times k_{j}}^{\left(j\right)}\right\}$, where *j *is the index of modules, and *k*_*j *_is the dimensionality of each respective module.

#### Step 3. Selecting latent factors that co-regulate genes with clinical factors

This step uses the original expression matrix ***G***.

(a) For the clinical factor set B* and every identified factor set ***F***^*(j)*^, find the associated genes. This is done by finding the projection length of each gene onto the subspace following *eq.2*, and finding the significance level using the *F *statistic,

(7) $$F=\frac{l^{2}}{k}\times \frac{n-k-1}{1-l^{2}}$$

where *l *is the projection length of the gene, *n *is the number of samples, and *k *is the dimensionality of the subspace. The test statistic follows the *F*_*k,n-k-1 *_distribution. The projection length and significance level are invariant to the rotation of the factors. We then transform the *F*-test p-value to false-discovery rate (FDR), and find the genes associated with each factor set at a certain FDR cutoff, e.g. 0.1.

(b) For every identified factor set ***F***^(*j*)^, test the overlap between its associated genes with the genes associated with the clinical factor set ***B***. The calculation takes into account of potential false positives. Assuming the total number of genes *is p*, the count of genes associated with ***B ***is *m*_1_, the count of genes associated with ***F***^(*j*) ^*is m*_2_, the count of overlapping genes is *r*, and the FDR cutoff is λ, we use $m_{1}^{\prime }=ceiling\left(m_{1}\left(1-\lambda \right)\right)$, $m_{2}^{\prime }=ceiling\left(m_{2}\left(1-\lambda \right)\right)$, and *r' *= *floor*(*r*(1 - *λ*)^2^) for the calculation of the hypergeometric p-value in a conservative manner:

(8) $$P=\sum_{l\geq r^{\prime }}\frac{\left(\begin{array}{c} p-m_{1}^{\prime }\\ {m^{\prime }}_{2}-l \end{array}\right)\left(\begin{array}{c} m_{1}^{\prime }\\ l \end{array}\right)}{\left(\begin{array}{c} p\\ m_{2}^{\prime } \end{array}\right)}$$

The overlap is called significant if *P *is smaller than a cutoff, e.g. 0.01.

(c) If an identified factor set shows significant gene overlapping with the clinical factor set, we further test each of its factors separately for gene overlapping with the clinical factor set, using the same strategy as in steps (a) and (b). Only significant factors are retained.

### Simulation study

We simulated data matrices with 2000 genes and 100 samples. Among the 2000 genes, module one of 200 genes were governed by the combination of a clinical factor and some other (1 to 3) factors. Four other modules of 200 genes were each governed by 2 to 4 factors that are independent from module one. All factor scores were independently drawn from the standard normal distribution. The remaining 1000 genes were pure noise genes. Three levels of measurement noise were simulated, with signal to noise ratio (S/N) equal to 0.5, 1, and 2.

Two versions of GLFD were tested, one with exhaustive factor search, the other with sequential factor search. Two methods were used as comparison. The first was partial least squares (PLS) regression [17]. PLS finds a subspace to project both the genes and the outcome variables, such that the projected genes explain the maximum multidimensional variance of the projected outcome. The latent factors defining the subspace were used in our comparison. The second method we compared was supervised principal components (SPC) [18]. SPC first extracts genes with first-order relations with the outcome, and then finds the principal components of the selected genes. The eigen vectors were taken as the latent factors identified by SPC. Two variants of SPC were used - (1) allowing the method to select the cutoff using cross-validation, and (2) using the true number of genes belonging to the module with the clinical factor. We note that neither PLS nor SPC is for latent factor discovery. Rather, both methods aim at predictive model building. Both PLS and SPC order the latent factors based on their contribution to the prediction of the clinical factor, and require user specification of the dimensionality of the subspace.

For a simulated data matrix with *k *true latent factors acting in combination with the clinical factor, we selected the first *k *latent factors found by each method. The selection was based on p-values for GLFD, and simply the first *k *factors for PLS and SPC. In the situation that GLFD found less than *k *latent factors at the p-value cutoff of 0.01, we used all the identified factors. In order to judge the effectiveness of the methods to recover the latent subspace, we examined how well each true latent factor was recovered. We used the multiple *R*^2 ^value of the regression of each true latent factor against the identified factors. At each parameter setting, the simulation was performed 100 times. The empirical distributions of the *R*^2 ^values were plotted and compared across the methods. The ideal method should yield multiple *R*^2 ^values close to one.

## Results and Discussions

### Simulation results

**Figure 1:**
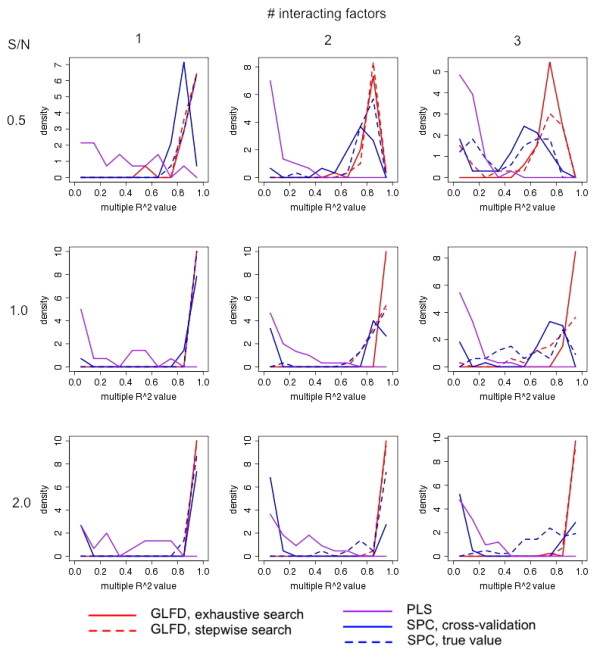
**Simulation results showing the capability of GLFD to discover latent factors, as compared with PLS and SPC**. In every simulation, 5 modules, each consisting of 200 simulated genes, were generated. The first module was governed by the clinical factor, together with 1~3 other latent factors (columns). The other four modules were governed by 2~4 factors. All factor scores were drawn independently from the standard normal distribution. Gaussian random noise was added to achieve different signal to noise ratios (rows). An additional 1000 pure noise genes were generated from the standard normal distribution. Each simulation setting was repeated 100 times. The success of latent factor recovery was evaluated by the R^2 ^values obtained by the regression of each latent factor against the identified factors. The relative frequencies (10 equal-sized bins between 0 and 1, equivalent to the histogram) of the R^2 ^values are plotted.

Figure 1 shows the simulation results. The two red curves represent GLFD (solid: exhaustive factor search; dashed: sequential factor search), and the two blue curves represent SPC (solid: cross validation - based cutoff selection; dashed: cutoff based on the true number of genes belonging to the module). By comparing the two red curves, we can see that GLFD using exhaustive factor search performed better than using sequential factor search in the original version of MLSA, which missed some latent factors when the signal to noise ratio was low. GLFD using exhaustive factor search clearly outperformed SPC, especially when more latent factors regulate the gene expression together with the clinical factor (right column). SPC extracts global structure given the set of genes associated with the outcome, and it uses hard cutoff to select such genes. GLFD extracts modular structure, and uses a model-based weighting scheme. In all simulation settings, PLS trailed other methods in terms of latent factor recovery. This is expected because PLS seeks subspaces that best predict the clinical outcome, while it may not be ideal for the purpose of finding factors acting in combinations with the clinical outcome. When the hidden truth was that no latent factor co-regulated genes with the clinical factor, the frequency of GLFD using exhaustive search identifying false-positive latent factors was 0.05 for S/N = 0.5, 0.03 for S/N = 1, and 0.06 for S/N = 2. For GLFD using stepwise search, the corresponding frequencies were 0, 0.02 and 0.06 respectively. The two methods being compared, SPC and PLS, do not have straight-forward criteria to determine the number of latent factors.

### Methotrexate treatment response in primary acute lymphoblastic leukemia (ALL) (GSE10255)

**Figure 2:**
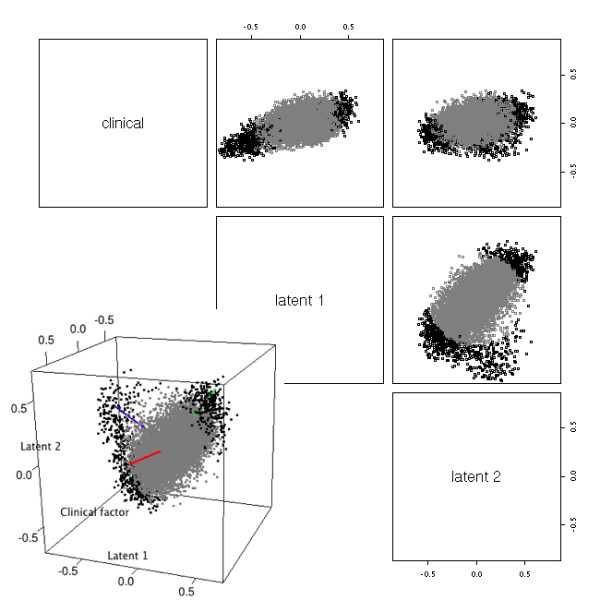
**Factors found from GSE10255 dataset**. Pair-wise scatterplots of the gene expression projected onto the clinical factor and the latent factors found by GLFD are shown. Black points correspond to genes with projection length > 0.4 onto the three dimensional subspace. Inset: three-dimensional plot of the genes' projection onto the subspace spanned by the clinical factor and the two latent factors. The axes were rotated using oblique rotation. The blue and green axes align with point clouds away from the origin.

Downloaded from the Gene Expression Omnibus (GEO) [19], The GSE10255 dataset is the gene expression in primary acute lymphoblastic leukemia (ALL) associated with methotrexate (MTX) treatment [20]. The major clinical outcome is the reduction of circulating leukemia cells after initial MTX treatment. We performed the analysis by GLFD and identified two latent factors that act in combination with the observed factor of MTX response. When we examined the scatter plots of the projection lengths of all genes onto the three factors (one clinical factor and two latent factors), some interesting patterns were observed (Figure 2): First, the projection length of genes onto the clinical factor is generally low, the maximum being 0.377. This indicates only a weak first-order transcriptional response is linked to the clinical response. Second, the projection of genes onto the clinical factor and the latent factors showed a clear pattern off the axes, indicating the transcriptional response is better interpreted as a combination of several components.

We show the projections of genes onto the 3D subspace spanned by the clinical factor and the latent factors (Figure 2, inset). We performed oblique rotation [21] on the genes with projection length > 0.4 on this 3D subspace. After rotation, the blue and green axes each aligned to a point cloud away from the origin. The red axis captures little information with regard to genes having large projection lengths on the 3D subspace. In order to shed light on the biological meaning of the blue and green components, we resorted to gene set analysis [3,22,23] of gene ontology biological processes [24]. To reduce the redundancy in GO and make the results easily interpretable, we used an organism-specific heuristic scheme to select a subset of GO biological process terms such that the selected terms were relatively specific, yet not too narrow [25]. Starting from the broad term "biological process", the method examined the number of human ENTREZ genes assigned to each GO term and its descendent terms. If over 40% of the term's genes (70% if the term has < 500 genes) were assigned to its descendent terms, the term was considered to be too broad and was replaced by its direct descendent terms. Otherwise the term was kept in the final selection. The method iteratively examined all biological process terms until it reached terms with < 5 genes assigned, which were ignored. A total of 803 GO biological process terms were selected, which covered 10420 ENTREZ genes. The minimum number of genes assigned to a selected term was 5, the maximum 1066, and the median 13.

We performed gene set analysis using the method GSA by Efron and Tibshirani [26], which handles continuous outcomes. We used the rotated factors themselves as the outcome variables in GSA in order to find gene sets that were significantly associated with them. Among the top 48 gene sets associated with the blue factor (*p *≤ 0.01), a large proportion (47.9%, compared to 4.6% among all gene sets under study) belonged to cell cycle/DNA metabolism - related processes (Additional file 1, Table S1). This is expected because the clinical factor itself is the reduction of circulating leukemia cells after MTX treatment. Among the top 37 gene sets associated with the green factor (*p *≤ 0.01), 18.9% of them were part of the immune system process, compared to 6.1% among all gene sets under study (Additional file 1, Table S2). This is consistent with MTX's function as an immunosuppressant [27]. In addition, 5 of the top 37 gene sets (13.5%, compared to 2.9% among all gene sets under study) were RNA metabolism/transport gene sets. It has been documented that the expression of RNA metabolism/transport genes tend to be altered in methotrexate-resistant cells [28].

Gene set analysis on the clinical factor itself showed enrichment of cell cycle/DNA metabolism gene sets among the top gene sets (28.8%, compared to 47.9% associated with the blue factor and 4.6% among all gene sets under study; Additional file 1, Table S3). Yet the immune system gene sets were no longer enriched in the list (5.8%, compared to 18.9% associated with green factor and 6.1% among all terms under study). Combined with the fact that the projection lengths of genes onto the clinical factor are relatively small (maximum is 0.377), we see that focusing only on genes/gene sets directly correlated with the clinical factor causes loss of power to detect significant gene expression changes in MTX response. GLFD was able to reveal hidden factors that act in combination with the clinical factor, and substantially enhance the data interpretation. In this dataset, the clinical factor is an observed outcome potentially with measurement errors. We can view the MTX response as a combination of several underlying molecular events, the strongest of which being biological processes related to cell reproduction and the immune system.

As a comparison, we also applied SPC and principal component analysis (PCA) on the dataset. PCA was included because of its popularity in practice. The clinical factor had weak impact on gene expression, hence weak correlation with the leading PCs identified by both SPC and PCA. For both methods, we performed oblique rotation using the clinical factor and the first two PCs (Additional file 1, Figures S1 ~ S4). Similar to the case of GLFD, the projections of genes onto the three-dimensional subspace were mostly explained by two latent factors. We then conducted gene set analysis by GSA on the two factors. The first latent factor found through SPC showed enrichment of cell cycle-related gene sets (18.4%, compared to 47.9% by GLFD and 4.6% among all gene sets under study; Additional file 1, Table S4). The second factor found through SPC showed enrichment of immune system gene sets (17%, compared to 18.9% by GLFD and 6.1% in all gene sets under study; Additional file 1, Table S5). Neither factor showed enrichment of RNA metabolism/transport gene sets (compared to 13.5% by GLFD and 2.9% among all gene sets understudy). The first latent factor found through PCA did not show clear enrichment of any major functional group (Additional file 1, Table S6). The second latent factor found through PCA showed enrichment of immune system gene sets (12.5%, compared to 18.9% by GLFD and 6.1% in all gene sets under study; Additional file 1, Table S7). Overall, GLFD showed a better performance on the GSE10255 dataset in terms of finding relevant functional groups.

### Triple negative breast cancers (TNBC) v.s. primary breast tumors representing all subtypes (GSE18864)

**Figure 3:**
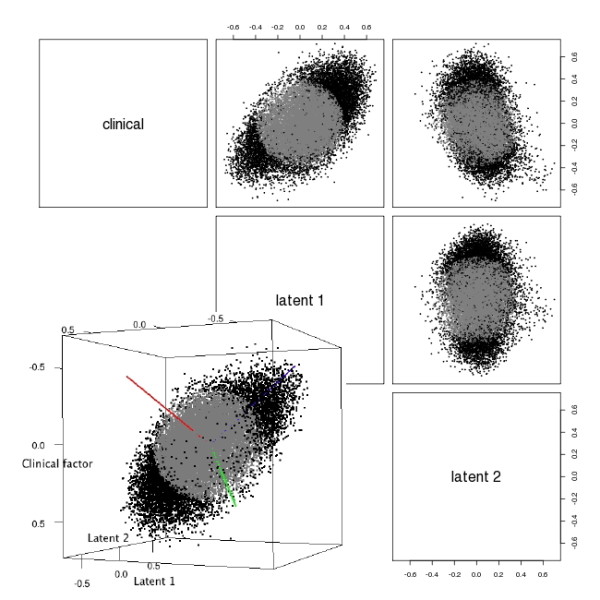
**Factors found from GSE18864 dataset**. Pair-wise scatterplots of the gene expression projected onto the clinical factor and the two latent factors found by GLFD are shown. Black points correspond to genes with projection length > 0.4 onto the three dimensional subspace. Inset: three-dimensional plot of the genes' projection onto the subspace spanned by the clinical factor and the two latent factors. The axes were rotated using PCA. Blue: first PC, green: second PC, red: third PC.

The second dataset we analyzed was the GSE18864 dataset [29], which compares the gene expression of 24 sporadic triple negative breast cancer (TNBC) samples against 51 primary breast tumor samples representing all subtypes. TNBC is characterized by the lack of expression of estrogen receptor (ER), progesterone receptor (PgR), and the human epidermal growth factor receptor 2 (ERBB2) [30]. GLFD identified two latent factors (Figure 3). As shown in the scatter plots, the shape of the point cloud in the three-dimensional subspace is close to elliptical. Thus we used principal component analysis on the projections of the genes with projection length > 0.4 on the three dimensional subspace. Rotated factors 1 (blue) and 2 (green) captured most of the information (Figure 3, inset).

We conducted GSA analysis to find gene sets significantly associated with the rotated axes. 17.9% of the gene sets associated with the blue axis (PC1) were signal transduction pathways (Additional file 1, Table S8), compared to 7.7% in all the gene sets under study. We examined the gene sets for their known links to TNBC and breast tumors in general, and found all of the seven gene sets to be associated with breast cancer phenotypes. Some of them have documented link to TNBC specifically. The G-protein coupled receptor protein signaling pathway (GO:0007186) involves GPR30 which modulates the progress of estrogen-related cancers [31]. Synaptophysin, which is a member of the synaptic transmission process (GO:0007268) and a marker of neuroendocrine (NE) differentiation, is important in breast cancer prognostics [32]. It is also one of the markers differentiating between basal-like breast cancer and triple negative breast cancer [33]. An associated term that doesn't belong to signal transduction, GO:0007416 synapse assembly, was also found to be significant. Nuclear factor of kappaB (NF-kappaB, member of GO:0043123) and its associated signaling pathway plays an important role in tumor development [34]. Among genes belonging to the biological process "signal complex assembly" (GO:0007172), filamin A is important in breast cancer cell migration [35], and Src is a potential treatment target for TNBC [36]. EGFR and EGFR ligands (member of GO:0007173) play a key role in breast cancer [37] and TNBC specifically [38]. Lower level of EGFR expression is associated reduced metastasis risk in TNBC [39]. JNK pathway (GO:0007254) modulates the anticancer effect of estradiol in human breast cancer cells [40]. RAB small GTPases (member of GO:0007264) were found to be genetically associated with breast cancer outcome [41]. Rho small GTPases and their effectors (member of GO:0007264) are known to affect the motility and metastasis of breast cancer cells [42]. In addition to the signal transduction gene sets, we also noticed three gene sets related to cell motility (GO:0007026, GO: 0007156, GO:0007018) were significant (Additional file 1, Table S8). This is consistent with the role of the two significant signal transduction pathways that are linked to cell motility in breast cancer (GO:0007172 and GO:0007264). In addition, the latent factor also showed association with cell cycle gene sets (12.8%, compared to 4.6% among all gene sets under study; Additional file 1, Table S8), which could be related to the different growth characteristics of TNBC [43].

Sixteen gene sets were significantly associated with the green axis (Additional file 1, Table S9), seven of which were immune/cytokine/stimulus response-related genet sets (43.8%, compared to 18.6% among all terms under study), excluding the "sleep" process. Study by immunohistochemistry has documented the loss of HLA class 1 in association with breast cancer and metastasis [44]. Interleukin 6 was found to be expressed in breast cancer tissues [45], and the blood concentration of IL6 is a negative prognosticator for breast cancer [46]. At a more general level, according to the Genes-to-Systems Breast Cancer (G2SBC) Database [47], a large number of stress response genes have altered expression in association with breast cancer.

Results from the GSA analysis on the clinical factor were far from as clear-cut as those from the rotated factors (Additional file 1, Table S10). The 17 significant gene sets included four (23.5%, compared to 18.6% overall) immune/cytokine/stimulus response genet sets, and two signal transduction gene sets (11.8%, compared to 7.7% overall). The clinical factor can be seen as a projection of a much stronger signal that's captured by the blue axis (Figure 3, inset).

As a comparison, we also conducted similar analysis by SPC and PCA. In this dataset, the clinical factor has a strong impact on gene expression. For both SPC and PCA, the subspace spanned by the first three PCs captured the clinical factor (multiple R^2 ^> 0.8). Thus we used the first three PCs for both methods, and performed factor rotation in the same manner as GLFD (Additional file 1, Figures S5 ~ S8). Unlike GLFD, the projections of genes onto the three dimensional subspace could not be explained by two latent factors. We performed gene set analysis using GSA on all three latent factors for both SPC and PCA. The first latent factor found through SPC didn't show clear enrichment of any major functional group (Additional file 1, Table S11). The second latent factor showed enrichment of cell cycle gene sets (26.5%, compared to 12.8% by GLFD and 4.6% among all gene sets under study; Additional file 1, Table S12), as well as slight enrichment of immune/cytokine/stimulus response-related genet sets (26.5%, compared to 43.8% by GLFD and 18.6% among all gene sets under study). The third latent factor showed enrichment of immune/cytokine/stimulus response-related genet sets (30.1%, compared to 43.8% by GLFD and 18.6% among all gene sets under study; Additional file 1, Table S13). None of the three factors showed enrichment of signaling pathways (compared to 17.9% by GLFD and 7.7% among all gene sets under study). For the factors found through PCA, only the second factor showed enrichment of cell cycle gene sets (30%, compared to 12.8% by GLFD and 4.6% among all gene sets under study; Additional file 1, Tables S14~S16). It is notable that the most prominent factor found by both SPC and PCA weren't clearly associated with any functional category. A possible explanation is that both methods captured vague global information in the data. In terms of finding relevant functional categories, SPC, which was competitive in some of the simulation settings, was close to GLFD, while PCA lagged behind.

In the real data analysis, we used two datasets that were generated from well-characterized diseases and treatment. The results confirmed the biological relevance of the findings by GLFD. In less well-characterized datasets, GLFD can help answer the question "What else has happened besides the differential expression?". The latent factors that GLFD seeks to identify are orthogonal to the clinical factors. This means they may not contribute to the prediction of the clinical outcome. However, in many situations, the goal of the study is to gain biological insight into the mechanisms of diseases. In addition, as demonstrated in the case of the MTX response data, the clinical outcome itself may be measured using a traditional marker, possibly with measurement error. In such situations, finding latent factors helps to better interpret the data and generate hypotheses of potential pathways that are activated together with the clinical outcome. GLFD uses weighted residuals of genes after projecting onto the clinical factors. Modular decomposition of a large matrix amounts to search in a very high dimensional space [15]. It is difficult computationally to reach the global optimum. The use of weighted residuals greatly reduces the search space by focusing the downstream steps on genes that are significantly associated with the clinical factors. In addition, it guarantees orthogonality between the identified factors and the clinical factors.

An alternative approach is to apply MLSA directly to the expression matrix, and then select factors that co-regulate genes with the clinical factor. We tested the idea on the two datasets. The post-processing became more involving as a much larger number of factors were identified, and they were not orthogonal to the clinical factors. We used a heuristic approach to address this issue. We forced the identified factors to be orthogonal to the clinical factor by subtracting their projection onto the clinical factor. We then applied the same factor selection procedure as in Step 3 of the Methods section. For both the GSE10255 dataset and the GSE18864 dataset, the alternative approach selected the same number of factors as GLFD. We applied the same rotation procedures for each dataset respectively as described above, and tested the latent factors for gene set association by GSA (Additional file 1, Figures S9 ~ S12). For the GSE10255 dataset, the first latent factor showed enrichment of immune system gene sets (20%, compared to 18.9% by GLFD and 6.1% among all gene sets under study; Additional file 1, Table S17), and the second latent factor showed enrichment of cell cycle gene sets (32.9%, compared to 47.9% by GLFD and 4.6% among all gene sets under study; Additional file 1, Table S18). Neither factor showed enrichment of RNA metabolism/transport gene sets (compared to 13.5% by GLFD and 2.9% among all gene sets under study). For the GSE18864 dataset, the first latent factor showed enrichment of cell cycle gene sets (31.4%, compared to 12.8% by GLFD and 4.6% among all gene sets under study; Additional file 1, Table S19), and the second latent factor showed enrichment of signaling gene sets (12.9%, compared to 17.9% by GLFD and 7.7% among all gene sets under study; Additional file 1, Table S20). Neither factor showed enrichment of immune/cytokine/stimulus response-related genet sets (compared to 43.8% by GLFD and 18.6% among all gene sets under study). The results of the comparisons showed that while the alternative approach required more post-processing, its performance was not as good as GLFD in terms of finding relevant functional categories.

The main purpose of GLFD is to identify the subspace governed by both the clinical factor(s) and latent factors. Genes showing large projections onto the subspace are considered to be in a clinically relevant module. For the latent factors to belong to the module, a significant number of the genes in the module need to be regulated by both the clinical factor(s) and the latent factors. In the search of latent factors, GLFD maintains the orthogonality between the observed clinical factor(s) and the latent factors, as well as between the latent factors. Once the subspace is determined, there are several ways to handle the factors - (1) keep the identified factors, (2) rotate the factors while maintaining orthogonality, (3) rotate the factors without maintaining orthogonality, and (4) rotate only the latent factors with/without orthogonality constraint. As the dimensionality is drastically reduced, the projection of the entire data onto the subspace can be visualized to help the user make a decision. Similar to the situation of traditional factor analysis, the choice of rotation depends on the data structure and user interpretation, which is beyond the scope of the GLFD method.

## Conclusions

In summary, we developed a new approach to interpret high throughput data and the associated algorithm based on modular matrix decomposition. The method is effective in bringing more insights into the data by finding latent factors that co-regulate genes with observed clinical factors. It can be used as an explorative tool for data interpretation and hypothesis generation.

## Authors' contributions

TY and YB jointly initiated the study and developed the hypotheses to be tested by the computational tools. TY developed the algorithms and performed data processing. YB carried out data interpretation. TY and YB jointly drafted the manuscript. All authors read and approved the final manuscript.

## Supplementary Material

Additional file 1 **Supplemental tables and figures**. Click here for file
