# Supplementary material for: Improving gene expression data interpretation by finding latent factors that co-regulate gene modules with clinical factors
Source: BMC Genomics. 2011 Nov 16;12:563. doi: 10.1186/1471-2164-12-563 (PMC3282832; doi:10.1186/1471-2164-12-563)
Supplement: Additional file 1 — Supplemental tables and figures. [file 1471-2164-12-563-S1.PDF]

Supporting Materials.

1. GSE10255.
1.1. GLFD results.

Table S1. Biological processes associated with rotated axis 1 (blue) (p≤0.01).

GOID	Term	pvalue
GO:0000077	DNA damage checkpoint	<5.00E-4
GO:0000079	regulation of cyclin-dependent protein kinase activity	<5.00E-4
GO:0000085	G2 phase of mitotic cell cycle	<5.00E-4
GO:0000086	G2/M transition of mitotic cell cycle	<5.00E-4
GO:0006260	DNA replication	<5.00E-4
GO:0006266	DNA ligation	<5.00E-4
GO:0006281	DNA repair	<5.00E-4
GO:0006310	DNA recombination	<5.00E-4
GO:0007018	microtubule-based movement	<5.00E-4
GO:0007051	spindle organization	<5.00E-4
GO:0007059	chromosome segregation	<5.00E-4
GO:0007067	mitosis	<5.00E-4
GO:0007507	heart development	<5.00E-4
GO:0009263	deoxyribonucleotide biosynthetic process	<5.00E-4
GO:0010212	response to ionizing radiation	<5.00E-4
GO:0030261	chromosome condensation	<5.00E-4
GO:0034508	centromere complex assembly	<5.00E-4
GO:0046605	regulation of centrosome cycle	<5.00E-4
GO:0048015	phosphoinositide-mediated signaling	<5.00E-4
GO:0051293	establishment of spindle localization	<5.00E-4
GO:0051298	centrosome duplication	<5.00E-4
GO:0051299	centrosome separation	<5.00E-4
GO:0051301	cell division	<5.00E-4
GO:0051303	establishment of chromosome localization	<5.00E-4
GO:0051323	metaphase	<5.00E-4
GO:0051436	negative regulation of ubiquitin-protein ligase activity involved in mitotic cell cycle	<5.00E-4
GO:0051437	positive regulation of ubiquitin-protein ligase activity involved in mitotic cell cycle	<5.00E-4
GO:0006334	nucleosome assembly	5.00E-04
GO:0009411	response to UV	5.00E-04
GO:0007126	meiosis	0.001
GO:0000082	G1/S transition of mitotic cell cycle	0.0015
GO:0006397	mRNA processing	0.0015
GO:0031503	protein complex localization	0.0015
GO:0007601	visual perception	0.002
GO:0009132	nucleoside diphosphate metabolic process	0.002
GO:0009219	pyrimidine deoxyribonucleotide metabolic process	0.0025
GO:0051028	mRNA transport	0.0025
GO:0008380	RNA splicing	0.0035

GO:0008654	phospholipid biosynthetic process	0.005
GO:0050772	positive regulation of axonogenesis	0.0055
GO:0050709	negative regulation of protein secretion	0.0065
GO:0007517	muscle organ development	0.007
GO:0009120	deoxyribonucleoside metabolic process	0.007
GO:0030183	B cell differentiation	0.007
GO:0044419	interspecies interaction between organisms	0.007
GO:0006954	inflammatory response	0.008
GO:0009156	ribonucleoside monophosphate biosynthetic process	0.009
GO:0007186	G-protein coupled receptor protein signaling pathway	0.0095

*Red terms: terms belonging to cell cycle/DNA metabolism – related processes; blue terms: terms belonging to the immune system; purple terms: RNA metabolism/transport terms.

Table S2. Biological processes associated with rotated axis 2 (green) (p≤0.01).

GOID	Term	pvalue
GO:0006397	mRNA processing	<5.00E-4
GO:0008380	RNA splicing	<5.00E-4
GO:0009060	aerobic respiration	<5.00E-4
GO:0009156	ribonucleoside monophosphate biosynthetic process	<5.00E-4
GO:0019882	antigen processing and presentation	<5.00E-4
GO:0042472	inner ear morphogenesis	<5.00E-4
GO:0006096	glycolysis	5.00E-04
GO:0045444	fat cell differentiation	5.00E-04
GO:0051436	negative regulation of ubiquitin-protein ligase activity involved in mitotic cell cycle	5.00E-04
GO:0051437	positive regulation of ubiquitin-protein ligase activity involved in mitotic cell cycle	0.001
GO:0042116	macrophage activation	0.0015
GO:0043368	positive T cell selection	0.0015
GO:0006144	purine base metabolic process	0.002
GO:0006919	activation of caspase activity	0.0025
GO:0002711	positive regulation of T cell mediated immunity	0.003
GO:0009120	deoxyribonucleoside metabolic process	0.003
GO:0043488	regulation of mRNA stability	0.004
GO:0045730	respiratory burst	0.004
GO:0050885	neuromuscular process controlling balance	0.004
GO:0007283	spermatogenesis	0.0045
GO:0007420	brain development	0.0045
GO:0030183	B cell differentiation	0.005
GO:0006108	malate metabolic process	0.0065
GO:0050852	T cell receptor signaling pathway	0.0065
GO:0051028	mRNA transport	0.007
GO:0006418	tRNA aminoacylation for protein translation	0.0075
GO:0009953	dorsal/ventral pattern formation	0.0075
GO:0055114	oxidation reduction	0.0075
GO:0006641	triglyceride metabolic process	0.008

GO:0006935	chemotaxis	0.008
GO:0009225	nucleotide-sugar metabolic process	0.008
GO:0015992	proton transport	0.008
GO:0051298	centrosome duplication	0.0085
GO:0032729	positive regulation of interferon-gamma production	0.009
GO:0014075	response to amine stimulus	0.01
GO:0048704	embryonic skeletal system morphogenesis	0.01
GO:0050853	B cell receptor signaling pathway	0.01

*Red terms: terms belonging to cell cycle/DNA metabolism – related processes; blue terms: terms belonging to the immune system; purple terms: RNA metabolism/transport terms.

Table S3. Biological processes associated with the clinical factor (p≤0.01).

GOID	Term	pvalue
GO:0000079	regulation of cyclin-dependent protein kinase activity	<5.00E-4
GO:0000085	G2 phase of mitotic cell cycle	<5.00E-4
GO:0001702	gastrulation with mouth forming second	<5.00E-4
GO:0006266	DNA ligation	<5.00E-4
GO:0007040	lysosome organization	<5.00E-4
GO:0007051	spindle organization	<5.00E-4
GO:0009263	deoxyribonucleotide biosynthetic process	<5.00E-4
GO:0030261	chromosome condensation	<5.00E-4
GO:0006281	DNA repair	5.00E-04
GO:0006310	DNA recombination	5.00E-04
GO:0006997	nucleus organization	5.00E-04
GO:0007059	chromosome segregation	5.00E-04
GO:0007507	heart development	5.00E-04
GO:0051293	establishment of spindle localization	5.00E-04
GO:0051436	negative regulation of ubiquitin-protein ligase activity involved in mitotic cell cycle	5.00E-04
GO:0007067	mitosis	0.001
GO:0042274	ribosomal small subunit biogenesis	0.001
GO:0051299	centrosome separation	0.001
GO:0007338	single fertilization	0.0015
GO:0051437	positive regulation of ubiquitin-protein ligase activity involved in mitotic cell cycle	0.0015
GO:0042326	negative regulation of phosphorylation	0.002
GO:0051301	cell division	0.002
GO:0019882	antigen processing and presentation	0.0025
GO:0006260	DNA replication	0.003
GO:0008206	bile acid metabolic process	0.003
GO:0008544	epidermis development	0.003
GO:0034405	response to fluid shear stress	0.003
GO:0032653	regulation of interleukin-10 production	0.0035
GO:0048754	branching morphogenesis of a tube	0.0035
GO:0007126	meiosis	0.004
GO:0030198	extracellular matrix organization	0.004

GO:0008217	regulation of blood pressure	0.0045
GO:0060037	pharyngeal system development	0.0045
GO:0006783	heme biosynthetic process	0.005
GO:0033005	positive regulation of mast cell activation	0.005
GO:0007018	microtubule-based movement	0.0055
GO:0031503	protein complex localization	0.0055
GO:0007160	cell-matrix adhesion	0.006
GO:0007586	digestion	0.006
GO:0020027	hemoglobin metabolic process	0.0065
GO:0006418	tRNA aminoacylation for protein translation	0.007
GO:0007186	G-protein coupled receptor protein signaling pathway	0.007
GO:0015844	monoamine transport	0.0075
GO:0048704	embryonic skeletal system morphogenesis	0.0075
GO:0006821	chloride transport	0.0085
GO:0032891	negative regulation of organic acid transport	0.0085
GO:0046131	pyrimidine ribonucleoside metabolic process	0.0085
GO:0009791	post-embryonic development	0.009
GO:0010212	response to ionizing radiation	0.009
GO:0002320	lymphoid progenitor cell differentiation	0.01
GO:0014047	glutamate secretion	0.01
GO:0048015	phosphoinositide-mediated signaling	0.01

*Red terms: terms belonging to cell cycle/DNA metabolism – related processes; blue terms: terms belonging to the immune system; purple terms: RNA metabolism/transport terms.

1.2. Supervised PCA results.

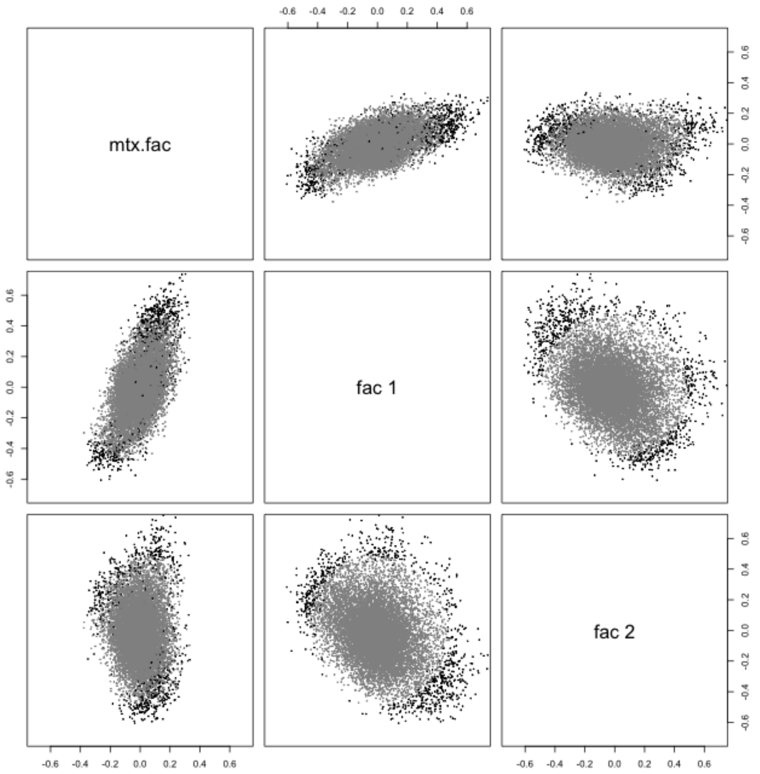

Figure S1. Pair-wise scatterplots of the gene expression projected onto the clinical factor and the two PCs found by SPC. Black points correspond to genes with projection length >0.4 onto the three dimensional subspace.

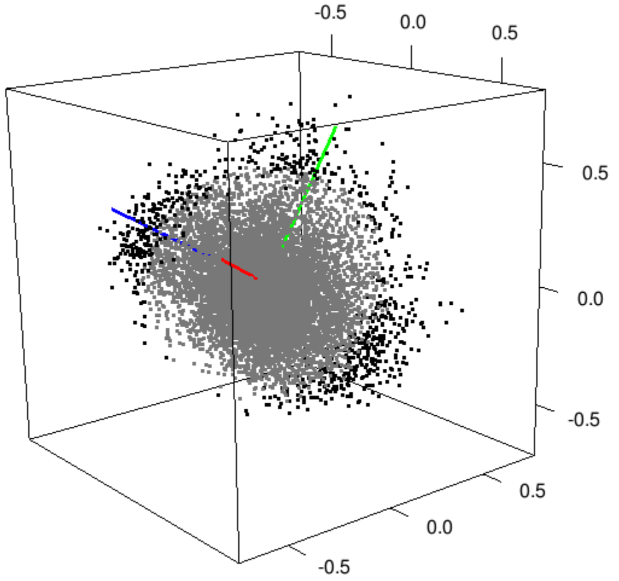

Figure S2. Three-dimensional plot of the genes' projection onto the subspace spanned by the clinical factor and the two PCs found by SPC. The colored axes were results of oblique rotation.

Table S4. Biological processes associated with rotated axis 1 (blue) (p≤0.01).

GOID	Term	pvalue
GO:0000085	G2 phase of mitotic cell cycle	<5.00E-4
GO:0002711	positive regulation of T cell mediated immunity	<5.00E-4
GO:0006511	ubiquitin-dependent protein catabolic process	<5.00E-4
GO:0007051	spindle organization	<5.00E-4
GO:0007160	cell-matrix adhesion	<5.00E-4
GO:0007186	G-protein coupled receptor protein signaling pathway	<5.00E-4
GO:0007268	synaptic transmission	<5.00E-4
GO:0007586	digestion	<5.00E-4
GO:0007601	visual perception	<5.00E-4
GO:0019882	antigen processing and presentation	<5.00E-4
GO:0043123	positive regulation of I-kappaB kinase/NF-kappaB cascade	<5.00E-4
GO:0044419	interspecies interaction between organisms	<5.00E-4
GO:0051293	establishment of spindle localization	<5.00E-4
GO:0051436	negative regulation of ubiquitin-protein ligase activity involved in mitotic cell cycle	<5.00E-4
GO:0051437	positive regulation of ubiquitin-protein ligase activity involved in mitotic cell cycle	<5.00E-4
GO:0006310	DNA recombination	5.00E-04
GO:0030183	B cell differentiation	5.00E-04
GO:0045730	respiratory burst	5.00E-04
GO:0051647	nucleus localization	5.00E-04
GO:0007608	sensory perception of smell	5.00E-04
GO:0008038	neuron recognition	5.00E-04
GO:0043010	camera-type eye development	5.00E-04
GO:0048704	embryonic skeletal system morphogenesis	5.00E-04
GO:0006919	activation of caspase activity	0.001
GO:0050853	B cell receptor signaling pathway	0.001

GO:0006936	muscle contraction	0.001
GO:0009952	anterior/posterior pattern formation	0.001
GO:0051303	establishment of chromosome localization	0.0015
GO:0006813	potassium ion transport	0.0015
GO:0007283	spermatogenesis	0.0015
GO:0007631	feeding behavior	0.0015
GO:0030855	epithelial cell differentiation	0.0015
GO:0051216	cartilage development	0.0015
GO:0006096	glycolysis	0.002
GO:0043368	positive T cell selection	0.002
GO:0051301	cell division	0.002
GO:0007156	homophilic cell adhesion	0.002
GO:0007588	excretion	0.002
GO:0006596	polyamine biosynthetic process	0.0025
GO:0007067	mitosis	0.0025
GO:0009263	deoxyribonucleotide biosynthetic process	0.0025
GO:0043193	positive regulation of gene-specific transcription	0.0025
GO:0007596	blood coagulation	0.003
GO:0032494	response to peptidoglycan	0.0035
GO:0006584	catecholamine metabolic process	0.0035
GO:0031128	developmental induction	0.0035
GO:0006401	RNA catabolic process	0.004
GO:0032729	positive regulation of interferon-gamma production	0.0045
GO:0043488	regulation of mRNA stability	0.0045
GO:0009953	dorsal/ventral pattern formation	0.0045
GO:0030326	embryonic limb morphogenesis	0.0045
GO:0007420	brain development	0.005
GO:0000082	G1/S transition of mitotic cell cycle	0.0055
GO:0045666	positive regulation of neuron differentiation	0.0055
GO:0019233	sensory perception of pain	0.006
GO:0006281	DNA repair	0.0065
GO:0009120	deoxyribonucleoside metabolic process	0.0065
GO:0016579	protein deubiquitination	0.0065
GO:0030261	chromosome condensation	0.0065
GO:0042116	macrophage activation	0.0065
GO:0050852	T cell receptor signaling pathway	0.0065
GO:0001541	ovarian follicle development	0.0065
GO:0048873	homeostasis of number of cells within a tissue	0.007
GO:0051299	centrosome separation	0.007
GO:0006334	nucleosome assembly	0.0075
GO:0000086	G2/M transition of mitotic cell cycle	0.008
GO:0046640	regulation of alpha-beta T cell proliferation	0.008
GO:0010873	positive regulation of cholesterol esterification	0.008
GO:0000079	regulation of cyclin-dependent protein kinase activity	0.0085
GO:0009060	aerobic respiration	0.0085

GO:0008544	epidermis development	0.0085
GO:0009408	response to heat	0.0085
GO:0001974	blood vessel remodeling	0.009
GO:0006836	neurotransmitter transport	0.009
GO:0070206	protein trimerization	0.0095
GO:0042475	odontogenesis of dentine-containing tooth	0.0095

*Red terms: terms belonging to cell cycle/DNA metabolism – related processes; blue terms: terms belonging to the immune system; purple terms: RNA metabolism/transport terms.

Table S5. Biological processes associated with rotated axis 2 (green) ($p \leq 0.01$).

GOID	Term	pvalue
GO:0006739	NADP metabolic process	<5.00E-4
GO:0006916	anti-apoptosis	<5.00E-4
GO:0006954	inflammatory response	<5.00E-4
GO:0007601	visual perception	<5.00E-4
GO:0016045	detection of bacterium	<5.00E-4
GO:0032490	detection of molecule of bacterial origin	<5.00E-4
GO:0032677	regulation of interleukin-8 production	<5.00E-4
GO:0042742	defense response to bacterium	<5.00E-4
GO:0046131	pyrimidine ribonucleoside metabolic process	<5.00E-4
GO:0007159	leukocyte cell-cell adhesion	5.00E-04
GO:0009953	dorsal/ventral pattern formation	5.00E-04
GO:0042119	neutrophil activation	5.00E-04
GO:0045087	innate immune response	5.00E-04
GO:0051260	protein homooligomerization	5.00E-04
GO:0006364	rRNA processing	0.001
GO:0007283	spermatogenesis	0.001
GO:0070231	T cell apoptosis	0.001
GO:0006465	signal peptide processing	0.0015
GO:0006935	chemotaxis	0.0015
GO:0006968	cellular defense response	0.0015
GO:0007420	brain development	0.0015
GO:0002891	positive regulation of immunoglobulin mediated immune response	0.002
GO:0045622	regulation of T-helper cell differentiation	0.002
GO:0060396	growth hormone receptor signaling pathway	0.002
GO:0007338	single fertilization	0.0025
GO:0002262	myeloid cell homeostasis	0.003
GO:0006897	endocytosis	0.003
GO:0007215	glutamate signaling pathway	0.003
GO:0009620	response to fungus	0.003
GO:0045730	respiratory burst	0.003
GO:0030855	epithelial cell differentiation	0.004
GO:0009791	post-embryonic development	0.006
GO:0032364	oxygen homeostasis	0.007
GO:0043123	positive regulation of I-kappaB kinase/NF-kappaB cascade	0.007

GO:0045123	cellular extravasation	0.007
GO:0051592	response to calcium ion	0.007
GO:0002446	neutrophil mediated immunity	0.008
GO:0007586	digestion	0.008
GO:0006879	cellular iron ion homeostasis	0.0085
GO:0006887	exocytosis	0.009
GO:0014047	glutamate secretion	0.009
GO:0042253	granulocyte macrophage colony-stimulating factor biosynthetic process	0.009
GO:0006401	RNA catabolic process	0.0095
GO:0014070	response to organic cyclic substance	0.0095
GO:0030036	actin cytoskeleton organization	0.0095
GO:0032091	negative regulation of protein binding	0.01
GO:0050857	positive regulation of antigen receptor-mediated signaling pathway	0.01

*Red terms: terms belonging to cell cycle/DNA metabolism – related processes; blue terms: terms belonging to the immune system; purple terms: RNA metabolism/transport terms.

1.3. PCA results.

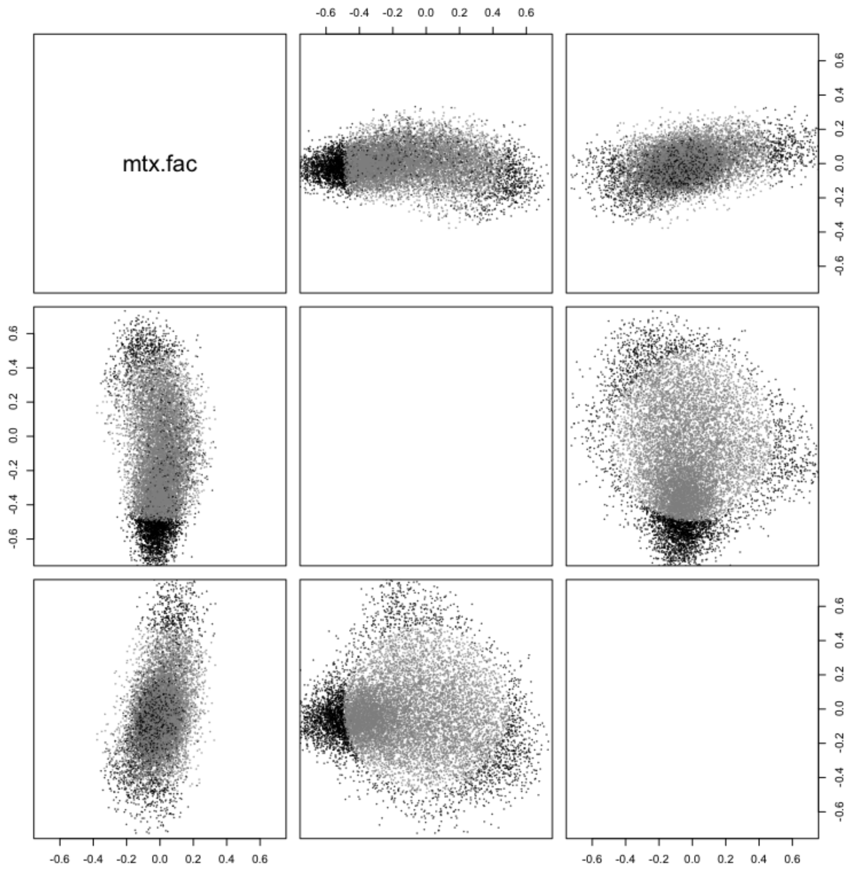

Figure S3. Pair-wise scatterplots of the gene expression projected onto the clinical factor and the two PCs found by PCA. Black points correspond to genes with projection length >0.4 onto the three dimensional subspace.

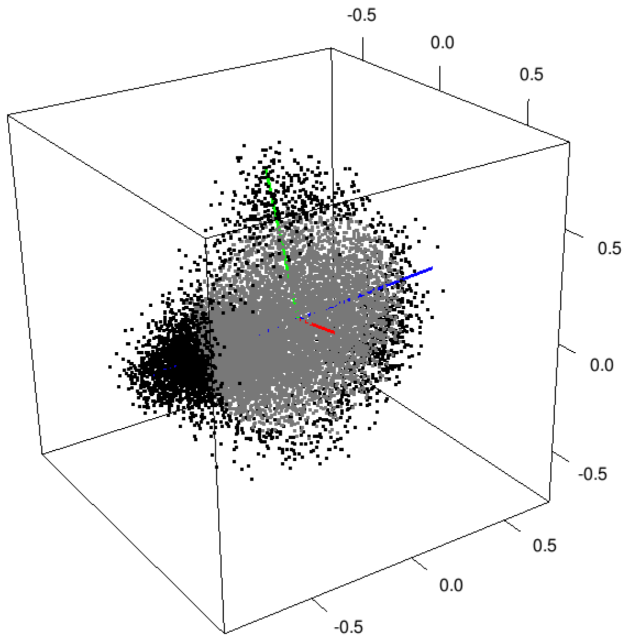

Figure S4. Three-dimensional plot of the genes' projection onto the subspace spanned by the clinical factor and the two PCs found by PCA. The colored axes were results of oblique rotation.

Table S6. Biological processes associated with rotated axis 1 (blue) (p≤0.01).

GOID	Term	pvalue
GO:0007156	homophilic cell adhesion	<5.00E-4
GO:0007186	G-protein coupled receptor protein signaling pathway	<5.00E-4
GO:0007608	sensory perception of smell	<5.00E-4
GO:0021515	cell differentiation in spinal cord	<5.00E-4
GO:0006281	DNA repair	5.00E-04
GO:0007268	synaptic transmission	5.00E-04
GO:0030252	growth hormone secretion	5.00E-04
GO:0043123	positive regulation of I-kappaB kinase/NF-kappaB cascade	5.00E-04
GO:0006813	potassium ion transport	0.001
GO:0007264	small GTPase mediated signal transduction	0.001
GO:0035272	exocrine system development	0.001
GO:0006821	chloride transport	0.0015
GO:0009615	response to virus	0.0015
GO:0043010	camera-type eye development	0.0015
GO:0007586	digestion	0.002
GO:0008016	regulation of heart contraction	0.002
GO:0008033	tRNA processing	0.002
GO:0009953	dorsal/ventral pattern formation	0.002
GO:0050852	T cell receptor signaling pathway	0.002
GO:0050909	sensory perception of taste	0.002
GO:0051881	regulation of mitochondrial membrane potential	0.002
GO:0006936	muscle contraction	0.0025
GO:0030198	extracellular matrix organization	0.0025
GO:0031645	negative regulation of neurological system process	0.0025

GO:0009954	proximal/distal pattern formation	0.003
GO:0048663	neuron fate commitment	0.003
GO:0008217	regulation of blood pressure	0.0035
GO:0019229	regulation of vasoconstriction	0.0035
GO:0051216	cartilage development	0.0035
GO:0030521	androgen receptor signaling pathway	0.004
GO:0042438	melanin biosynthetic process	0.004
GO:0006836	neurotransmitter transport	0.0045
GO:0016567	protein ubiquitination	0.0045
GO:0060350	endochondral bone morphogenesis	0.0045
GO:0002320	lymphoid progenitor cell differentiation	0.005
GO:0006360	transcription from RNA polymerase I promoter	0.005
GO:0006511	ubiquitin-dependent protein catabolic process	0.005
GO:0006886	intracellular protein transport	0.005
GO:0030855	epithelial cell differentiation	0.005
GO:0006024	glycosaminoglycan biosynthetic process	0.0055
GO:0016579	protein deubiquitination	0.0055
GO:0048754	branching morphogenesis of a tube	0.0055
GO:0050828	regulation of liquid surface tension	0.006
GO:0006783	heme biosynthetic process	0.0065
GO:0007030	Golgi organization	0.0065
GO:0050709	negative regulation of protein secretion	0.0065
GO:0001525	angiogenesis	0.007
GO:0006816	calcium ion transport	0.007
GO:0008544	epidermis development	0.007
GO:0021954	central nervous system neuron development	0.007
GO:0043616	keratinocyte proliferation	0.007
GO:0046887	positive regulation of hormone secretion	0.007
GO:0060004	reflex	0.007
GO:0006874	cellular calcium ion homeostasis	0.0075
GO:0030335	positive regulation of cell migration	0.0075
GO:0046677	response to antibiotic	0.0075
GO:0007411	axon guidance	0.008
GO:0017144	drug metabolic process	0.008
GO:0030879	mammary gland development	0.008
GO:0044419	interspecies interaction between organisms	0.008
GO:0048015	phosphoinositide-mediated signaling	0.0085
GO:0008038	neuron recognition	0.009
GO:0009083	branched chain family amino acid catabolic process	0.009
GO:0044253	positive regulation of multicellular organismal metabolic process	0.009

*Red terms: terms belonging to cell cycle/DNA metabolism – related processes; blue terms: terms belonging to the immune system; purple terms: RNA metabolism/transport terms.

Table S7. Biological processes associated with rotated axis 2 (green) (p≤0.01).

GOID	Term	pvalue
GO:0006936	muscle contraction	<5.00E-4
GO:0007186	G-protein coupled receptor protein signaling pathway	<5.00E-4
GO:0007268	synaptic transmission	<5.00E-4
GO:0008299	isoprenoid biosynthetic process	<5.00E-4
GO:0030574	collagen catabolic process	<5.00E-4
GO:0030855	epithelial cell differentiation	<5.00E-4
GO:0043368	positive T cell selection	<5.00E-4
GO:0045730	respiratory burst	<5.00E-4
GO:0050852	T cell receptor signaling pathway	<5.00E-4
GO:0002711	positive regulation of T cell mediated immunity	5.00E-04
GO:0006813	potassium ion transport	5.00E-04
GO:0006935	chemotaxis	5.00E-04
GO:0007160	cell-matrix adhesion	5.00E-04
GO:0007586	digestion	5.00E-04
GO:0007601	visual perception	5.00E-04
GO:0019882	antigen processing and presentation	5.00E-04
GO:0050853	B cell receptor signaling pathway	5.00E-04
GO:0006096	glycolysis	0.001
GO:0007156	homophilic cell adhesion	0.001
GO:0045666	positive regulation of neuron differentiation	0.001
GO:0050857	positive regulation of antigen receptor-mediated signaling pathway	0.001
GO:0051216	cartilage development	0.001
GO:0043123	positive regulation of I-kappaB kinase/NF-kappaB cascade	0.0015
GO:0048873	homeostasis of number of cells within a tissue	0.0015
GO:0007283	spermatogenesis	0.002
GO:0030198	extracellular matrix organization	0.002
GO:0031128	developmental induction	0.002
GO:0032109	positive regulation of response to nutrient levels	0.002
GO:0044419	interspecies interaction between organisms	0.002
GO:0007588	excretion	0.0025
GO:0030183	B cell differentiation	0.0025
GO:0050655	dermatan sulfate proteoglycan metabolic process	0.0025
GO:0051437	positive regulation of ubiquitin-protein ligase activity involved in mitotic cell cycle	0.0025
GO:0007608	sensory perception of smell	0.003
GO:0009225	nucleotide-sugar metabolic process	0.003
GO:0006401	RNA catabolic process	0.0035
GO:0006457	protein folding	0.0035
GO:0006310	DNA recombination	0.004
GO:0006783	heme biosynthetic process	0.004
GO:0008637	apoptotic mitochondrial changes	0.004
GO:0000085	G2 phase of mitotic cell cycle	0.0045
GO:0006919	activation of caspase activity	0.0045
GO:0043010	camera-type eye development	0.0045
GO:0055114	oxidation reduction	0.0045

GO:0006511	ubiquitin-dependent protein catabolic process	0.0055
GO:0009953	dorsal/ventral pattern formation	0.0055
GO:0043243	positive regulation of protein complex disassembly	0.0055
GO:0048704	embryonic skeletal system morphogenesis	0.006
GO:0007631	feeding behavior	0.0065
GO:0006584	catecholamine metabolic process	0.007
GO:0006836	neurotransmitter transport	0.007
GO:0008016	regulation of heart contraction	0.007
GO:0032364	oxygen homeostasis	0.007
GO:0051028	mRNA transport	0.007
GO:0070206	protein trimerization	0.007
GO:0045176	apical protein localization	0.0075
GO:0008038	neuron recognition	0.008
GO:0033631	cell-cell adhesion mediated by integrin	0.008
GO:0046112	nucleobase biosynthetic process	0.008
GO:0050909	sensory perception of taste	0.008
GO:0045408	regulation of interleukin-6 biosynthetic process	0.0085
GO:0009952	anterior/posterior pattern formation	0.009
GO:0006839	mitochondrial transport	0.0095
GO:0045582	positive regulation of T cell differentiation	0.01

*Red terms: terms belonging to cell cycle/DNA metabolism – related processes; blue terms: terms belonging to the immune system; purple terms: RNA metabolism/transport terms.

2. GSE18864.
2.1. GLFD results.

Table S8. Biological processes associated with rotated axis 1 (blue) ($p \leq 0.01$).

GOID	Term*	pvalue
GO:0007186	G-protein coupled receptor protein signaling pathway	<5.00E-4
GO:0007268	synaptic transmission	<5.00E-4
GO:0043123	positive regulation of I-kappaB kinase/NF-kappaB cascade	<5.00E-4
GO:0051647	nucleus localization	<5.00E-4
GO:0007051	spindle organization	5.00E-04
GO:0007067	mitosis	5.00E-04
GO:0007586	digestion	0.001
GO:0043550	regulation of lipid kinase activity	0.001
GO:0051301	cell division	0.001
GO:0000002	mitochondrial genome maintenance	0.0015
GO:0006836	neurotransmitter transport	0.0015
GO:0007283	spermatogenesis	0.0015
GO:0007608	sensory perception of smell	0.0015
GO:0042274	ribosomal small subunit biogenesis	0.0015
GO:0007026	negative regulation of microtubule depolymerization	0.0025
GO:0007605	sensory perception of sound	0.0025
GO:0051299	centrosome separation	0.0025
GO:0007172	signal complex assembly	0.003
GO:0010149	senescence	0.0035
GO:0006936	muscle contraction	0.004
GO:0050909	sensory perception of taste	0.004
GO:0042311	vasodilation	0.0045
GO:0007173	epidermal growth factor receptor signaling pathway	0.005
GO:0009296	flagellum assembly	0.005
GO:0009299	mRNA transcription	0.0055
GO:0007156	homophilic cell adhesion	0.006
GO:0007018	microtubule-based movement	0.0065
GO:0006744	ubiquinone biosynthetic process	0.007
GO:0043525	positive regulation of neuron apoptosis	0.007
GO:0015781	pyrimidine nucleotide-sugar transport	0.0075
GO:0006833	water transport	0.008
GO:0007596	blood coagulation	0.008
GO:0007254	JNK cascade	0.0085
GO:0007416	synapse assembly	0.0085
GO:0008217	regulation of blood pressure	0.0085
GO:0042384	cilium assembly	0.009
GO:0006584	catecholamine metabolic process	0.01
GO:0007264	small GTPase mediated signal transduction	0.01
GO:0045786	negative regulation of cell cycle	0.01

*Green terms: signal transduction terms; red terms: terms belonging to cell cycle/DNA metabolism – related processes; blue terms: terms belonging to the immune system/stimulus response/cytokine production.

Table S9. Biological processes associated with rotated axis 2 (green) (p≤0.01).

GOID	Term*	pvalue
GO:0042274	ribosomal small subunit biogenesis	<5.00E-4
GO:0006414	translational elongation	5.00E-04
GO:0006929	substrate-bound cell migration	5.00E-04
GO:0015711	organic anion transport	5.00E-04
GO:0032202	telomere assembly	5.00E-04
GO:0019882	antigen processing and presentation	0.0015
GO:0042384	cilium assembly	0.0035
GO:0048535	lymph node development	0.0045
GO:0006959	humoral immune response	0.006
GO:0030431	sleep	0.006
GO:0055114	oxidation reduction	0.006
GO:0031668	cellular response to extracellular stimulus	0.0065
GO:0043618	regulation of transcription from RNA polymerase II promoter in response to stress	0.007
GO:0030198	extracellular matrix organization	0.0095
GO:0045408	regulation of interleukin-6 biosynthetic process	0.01
GO:0050857	positive regulation of antigen receptor-mediated signaling pathway	0.01

*Green terms: signal transduction terms; red terms: terms belonging to cell cycle/DNA metabolism – related processes; blue terms: terms belonging to the immune system/stimulus response/cytokine production.

Table S10. Biological processes associated with the clinical factor (p≤0.01).

GOID	Term*	pvalue
GO:0007018	microtubule-based movement	5.00E-04
GO:0032722	positive regulation of chemokine production	5.00E-04
GO:0007264	small GTPase mediated signal transduction	0.001
GO:0022614	membrane to membrane docking	0.001
GO:0007026	negative regulation of microtubule depolymerization	0.0015
GO:0045080	positive regulation of chemokine biosynthetic process	0.002
GO:0007586	digestion	0.0035
GO:0032891	negative regulation of organic acid transport	0.0035
GO:0045123	cellular extravasation	0.0045
GO:0045815	positive regulation of gene expression, epigenetic	0.0045
GO:0006865	amino acid transport	0.0055
GO:0007190	activation of adenylate cyclase activity	0.0065
GO:0051293	establishment of spindle localization	0.0065
GO:0035313	wound healing, spreading of epidermal cells	0.008
GO:0048384	retinoic acid receptor signaling pathway	0.008
GO:0006641	triglyceride metabolic process	0.0085
GO:0046473	phosphatidic acid metabolic process	0.01

*Green terms: signal transduction terms; red terms: terms belonging to cell cycle/DNA metabolism – related processes; blue terms: terms belonging to the immune system/stimulus response/cytokine production.

2.2. SPC results.

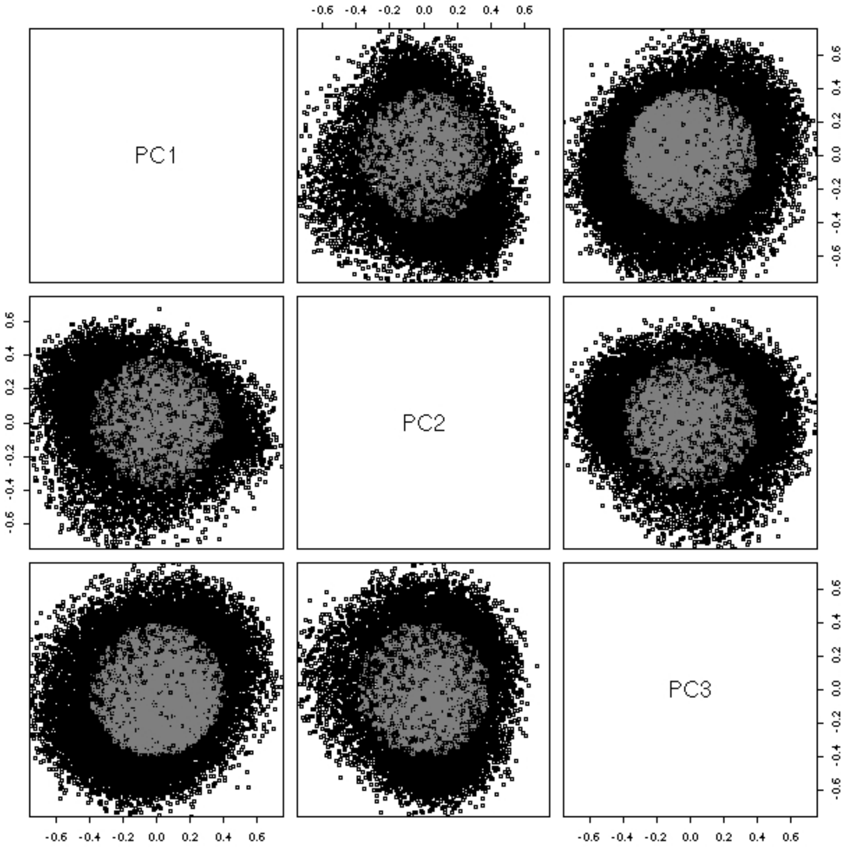

Figure S5. Pair-wise scatterplots of the gene expression projected onto the first three PCs found by SPC. PC1 is highly correlated with the clinical factor (correlation coefficient = 0.845). Black points correspond to genes with projection length >0.4 onto the three dimensional subspace.

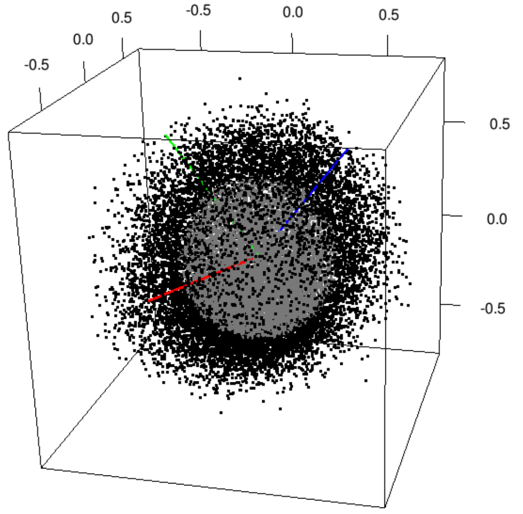

Figure S6. Three-dimensional plot of the genes' projection onto the subspace spanned by the first three PCs found by SPC. The colored axes were results of oblique rotation. The axes were rotated using PCA based only on the points that have projection length of >0.4.

Table S11. Biological processes associated with the rotated axis 1 (blue) (p≤0.01).

GOID	Term	pvalue
GO:0006935	sodium ion transport	<5.00E-4

GO:0016568	mRNA transcription	<5.00E-4
GO:0030259	heparan sulfate proteoglycan biosynthetic process	<5.00E-4
GO:0006342	gluconeogenesis	0.001
GO:0006739	glycoprotein catabolic process	0.0015
GO:0035137	adrenal gland development	0.0015
GO:0042311	regulation of vitamin metabolic process	0.0015
GO:0006903	oxygen and reactive oxygen species metabolic process	0.002
GO:0007156	intra-Golgi vesicle-mediated transport	0.002
GO:0060155	regulation of interleukin-6 biosynthetic process	0.002
GO:0018205	response to cold	0.0025
GO:0030539	nucleoside transport	0.0025
GO:0006684	protein amino acid sulfation	0.003
GO:0006468	DNA topological change	0.0035
GO:0019439	anterior/posterior pattern formation	0.0035
GO:0042742	cellular response to extracellular stimulus	0.0035
GO:0006570	translational elongation	0.004
GO:0007186	inflammatory response	0.0045
GO:0043496	response to lipopolysaccharide	0.0045
GO:0043550	regulation of interleukin-10 production	0.0045
GO:0051647	interspecies interaction between organisms	0.0045
GO:0030224	regulation of smooth muscle cell migration	0.005
GO:0006693	protein amino acid glycosylation	0.0055
GO:0006897	porphyrin catabolic process	0.0055
GO:0007172	substrate-bound cell migration	0.0055
GO:0006805	glycine metabolic process	0.006
GO:0033198	actin cytoskeleton organization	0.006
GO:0006890	ATP biosynthetic process	0.0065
GO:0016197	pyrimidine ribonucleotide biosynthetic process	0.0065
GO:0008354	synaptic transmission	0.0085
GO:0016045	nucleoside catabolic process	0.0085
GO:0051972	positive regulation of interleukin-12 biosynthetic process	0.009

*Green terms: signal transduction terms; red terms: terms belonging to cell cycle/DNA metabolism – related processes; blue terms: terms belonging to the immune system/stimulus response/cytokine production.

Table S12. Biological processes associated with the rotated axis 2 (green) (p≤0.01).

GOID	Term	pvalue
GO:0006281	DNA repair	<5.00E-4
GO:0007059	chromosome segregation	<5.00E-4
GO:0030032	lamellipodium assembly	<5.00E-4
GO:0030261	chromosome condensation	<5.00E-4
GO:0051303	establishment of chromosome localization	<5.00E-4
GO:0007051	spindle organization	5.00E-04
GO:0051293	establishment of spindle localization	5.00E-04

GO:0006954	inflammatory response	0.001
GO:0019882	antigen processing and presentation	0.001
GO:0045087	innate immune response	0.001
GO:0030101	natural killer cell activation	0.0015
GO:0007018	microtubule-based movement	0.002
GO:0051299	centrosome separation	0.002
GO:0051301	cell division	0.002
GO:0006968	cellular defense response	0.003
GO:0000085	G2 phase of mitotic cell cycle	0.0035
GO:0007067	mitosis	0.0035
GO:0010149	senescence	0.0035
GO:0006541	glutamine metabolic process	0.004
GO:0050857	positive regulation of antigen receptor-mediated signaling pathway	0.004
GO:0007411	axon guidance	0.0045
GO:0007631	feeding behavior	0.005
GO:0006935	chemotaxis	0.0055
GO:0034508	centromere complex assembly	0.0055
GO:0001701	in utero embryonic development	0.006
GO:0042953	lipoprotein transport	0.006
GO:0030033	microvillus assembly	0.0065
GO:0031047	gene silencing by RNA	0.0065
GO:0006397	mRNA processing	0.007
GO:0005977	glycogen metabolic process	0.008
GO:0006401	RNA catabolic process	0.008
GO:0008380	RNA splicing	0.0085
GO:0051028	mRNA transport	0.0095
GO:0035272	exocrine system development	0.01

*Green terms: signal transduction terms; red terms: terms belonging to cell cycle/DNA metabolism – related processes; blue terms: terms belonging to the immune system/stimulus response/cytokine production.

Table S13. Biological processes associated with the rotated axis 3 (red) (p≤0.01).

GOID	Term	pvalue
GO:0005977	glycogen metabolic process	<5.00E-4
GO:0006144	purine base metabolic process	<5.00E-4
GO:0006935	chemotaxis	<5.00E-4
GO:0006936	muscle contraction	<5.00E-4
GO:0007156	homophilic cell adhesion	<5.00E-4
GO:0007159	leukocyte cell-cell adhesion	<5.00E-4
GO:0007268	synaptic transmission	<5.00E-4
GO:0007283	spermatogenesis	<5.00E-4
GO:0009120	deoxyribonucleoside metabolic process	<5.00E-4
GO:0009156	ribonucleoside monophosphate biosynthetic process	<5.00E-4
GO:0009612	response to mechanical stimulus	<5.00E-4

GO:0019882	antigen processing and presentation	<5.00E-4
GO:0044419	interspecies interaction between organisms	<5.00E-4
GO:0045084	positive regulation of interleukin-12 biosynthetic process	<5.00E-4
GO:0050655	dermatan sulfate proteoglycan metabolic process	<5.00E-4
GO:0050853	B cell receptor signaling pathway	<5.00E-4
GO:0050857	positive regulation of antigen receptor-mediated signaling pathway	<5.00E-4
GO:0032964	collagen biosynthetic process	5.00E-04
GO:0048663	neuron fate commitment	5.00E-04
GO:0050852	T cell receptor signaling pathway	5.00E-04
GO:0060325	face morphogenesis	5.00E-04
GO:0006954	inflammatory response	0.001
GO:0007420	brain development	0.001
GO:0031579	membrane raft organization	0.001
GO:0043368	positive T cell selection	0.001
GO:0046112	nucleobase biosynthetic process	0.001
GO:0006959	humoral immune response	0.0015
GO:0007601	visual perception	0.0015
GO:0060558	regulation of calcidiol 1-monooxygenase activity	0.0015
GO:0008284	positive regulation of cell proliferation	0.002
GO:0008544	epidermis development	0.002
GO:0045582	positive regulation of T cell differentiation	0.002
GO:0046173	polyol biosynthetic process	0.002
GO:0046640	regulation of alpha-beta T cell proliferation	0.002
GO:0002698	negative regulation of immune effector process	0.0025
GO:0042088	T-helper 1 type immune response	0.0025
GO:0048469	cell maturation	0.0025
GO:0006917	induction of apoptosis	0.003
GO:0045087	innate immune response	0.003
GO:0006813	potassium ion transport	0.0035
GO:0032755	positive regulation of interleukin-6 production	0.0035
GO:0043123	positive regulation of I-kappaB kinase/NF-kappaB cascade	0.0035
GO:0007229	integrin-mediated signaling pathway	0.004
GO:0007586	digestion	0.004
GO:0009953	dorsal/ventral pattern formation	0.004
GO:0034097	response to cytokine stimulus	0.004
GO:0007338	single fertilization	0.0045
GO:0032729	positive regulation of interferon-gamma production	0.0045
GO:0050909	sensory perception of taste	0.0045
GO:0019722	calcium-mediated signaling	0.005
GO:0032653	regulation of interleukin-10 production	0.0055
GO:0009164	nucleoside catabolic process	0.007
GO:0022614	membrane to membrane docking	0.007
GO:0042446	hormone biosynthetic process	0.0075
GO:0019079	viral genome replication	0.008
GO:0042982	amyloid precursor protein metabolic process	0.0085

GO:0048708	astrocyte differentiation	0.0085
GO:0051145	smooth muscle cell differentiation	0.0085
GO:0042273	ribosomal large subunit biogenesis	0.009
GO:0045665	negative regulation of neuron differentiation	0.009
GO:0008633	activation of pro-apoptotic gene products	0.0095
GO:0006836	neurotransmitter transport	0.01
GO:0030855	epithelial cell differentiation	0.01

*Green terms: signal transduction terms; red terms: terms belonging to cell cycle/DNA metabolism – related processes; blue terms: terms belonging to the immune system/stimulus response/cytokine production.

2.3. PCA results.

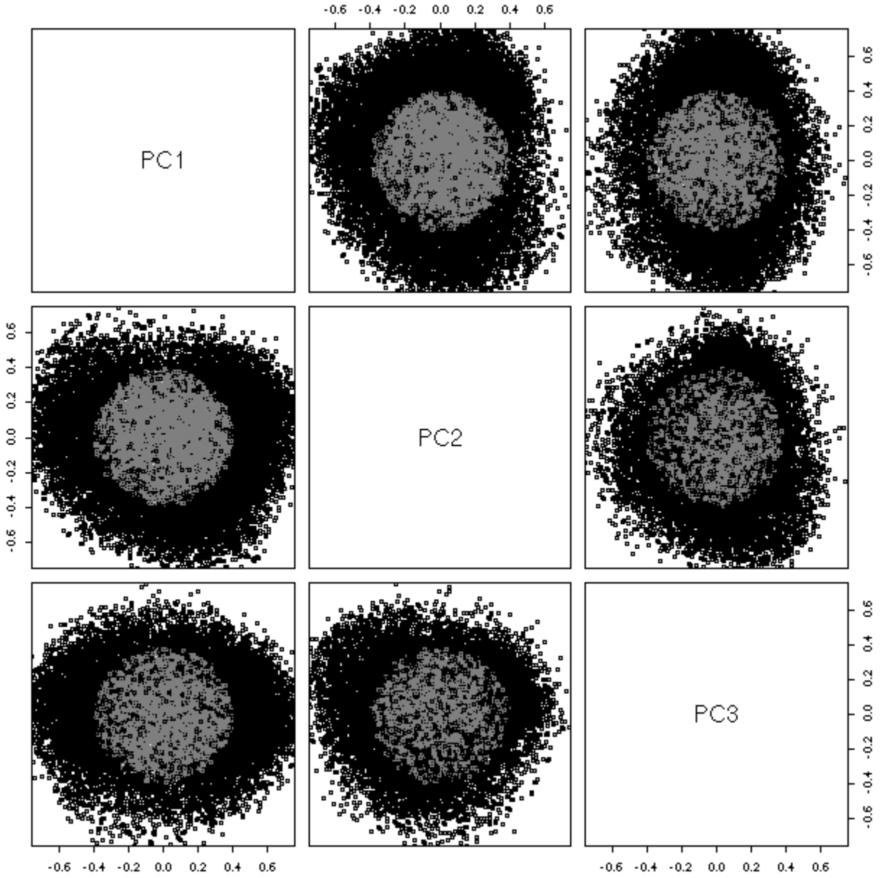

Figure S7. Pair-wise scatterplots of the gene expression projected onto the first three PCs found by SPC. The clinical factor mostly falls into the subspace (multiple R^2 = 0.80). Black points correspond to genes with projection length >0.4 onto the three dimensional subspace.

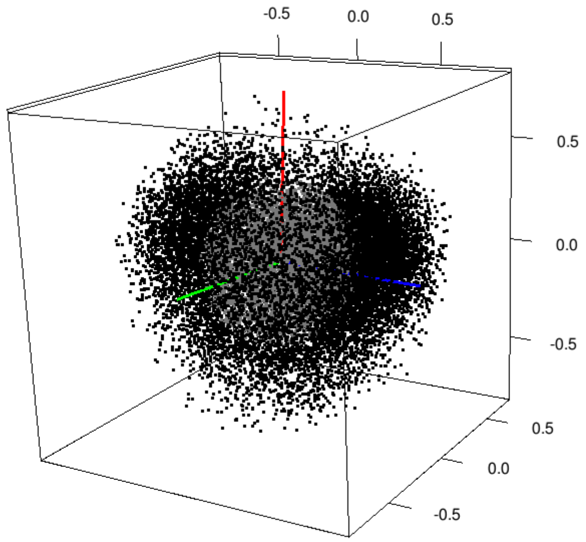

Figure S8. Three-dimensional plot of the genes' projection onto the subspace spanned by the first three PCs found by SPC. The colored axes were results of oblique rotation. The axes were rotated using PCA based only on the points that have projection length of >0.4.

Table S14. Biological processes associated with the rotated axis 1 (blue) (p≤0.01).

GOID	Term	pvalue
GO:0030259	lipid glycosylation	<5.00E-4
GO:0006468	protein amino acid phosphorylation	5.00E-04
GO:0055114	oxidation reduction	5.00E-04
GO:0006342	chromatin silencing	0.001
GO:0051647	nucleus localization	0.001
GO:0009156	ribonucleoside monophosphate biosynthetic process	0.0015
GO:0050771	negative regulation of axonogenesis	0.002
GO:0006414	translational elongation	0.0035
GO:0019439	aromatic compound catabolic process	0.004
GO:0032801	receptor catabolic process	0.005
GO:0006693	prostaglandin metabolic process	0.0055
GO:0016568	chromatin modification	0.0055
GO:0016339	calcium-dependent cell-cell adhesion	0.0065
GO:0006970	response to osmotic stress	0.007
GO:0008354	germ cell migration	0.007
GO:0006809	nitric oxide biosynthetic process	0.0075
GO:0060052	neurofilament cytoskeleton organization	0.0075
GO:0060037	pharyngeal system development	0.0085

*Green terms: signal transduction terms; red terms: terms belonging to cell cycle/DNA metabolism –related processes; blue terms: terms belonging to the immune system/stimulus response/cytokine production.

Table S15. Biological processes associated with the rotated axis 2 (green) (p≤0.01).

GOID	Term	pvalue
GO:0000085	G2 phase of mitotic cell cycle	<5.00E-4
GO:0006260	DNA replication	<5.00E-4

GO:0007018	microtubule-based movement	<5.00E-4
GO:0007051	spindle organization	<5.00E-4
GO:0030261	chromosome condensation	<5.00E-4
GO:0051293	establishment of spindle localization	<5.00E-4
GO:0051299	centrosome separation	<5.00E-4
GO:0051303	establishment of chromosome localization	<5.00E-4
GO:0006144	purine base metabolic process	5.00E-04
GO:0006281	DNA repair	5.00E-04
GO:0006541	glutamine metabolic process	5.00E-04
GO:0007059	chromosome segregation	5.00E-04
GO:0010149	senescence	5.00E-04
GO:0034508	centromere complex assembly	5.00E-04
GO:0007067	mitosis	0.001
GO:0051301	cell division	0.001
GO:0005977	glycogen metabolic process	0.0015
GO:0007157	heterophilic cell-cell adhesion	0.0015
GO:0006401	RNA catabolic process	0.002
GO:0006954	inflammatory response	0.002
GO:0045087	innate immune response	0.002
GO:0051323	metaphase	0.002
GO:0009067	aspartate family amino acid biosynthetic process	0.0025
GO:0051096	positive regulation of helicase activity	0.0025
GO:0051028	mRNA transport	0.003
GO:0008380	RNA splicing	0.0035
GO:0006821	chloride transport	0.004
GO:0035272	exocrine system development	0.004
GO:0006397	mRNA processing	0.0045
GO:0009156	ribonucleoside monophosphate biosynthetic process	0.0045
GO:0006874	cellular calcium ion homeostasis	0.0055
GO:0006730	one-carbon metabolic process	0.0065
GO:0016042	lipid catabolic process	0.007
GO:0032801	receptor catabolic process	0.0075
GO:0050909	sensory perception of taste	0.008
GO:0043666	regulation of phosphoprotein phosphatase activity	0.0085
GO:0007411	axon guidance	0.009
GO:0043094	cellular metabolic compound salvage	0.009
GO:0006265	DNA topological change	0.0095
GO:0006108	malate metabolic process	0.01

*Green terms: signal transduction terms; red terms: terms belonging to cell cycle/DNA metabolism – related processes; blue terms: terms belonging to the immune system/stimulus response/cytokine production.

Table S16. Biological processes associated with the rotated axis 3 (red) ($p \le 0.01$).

GOID	Term	pvalue

GO:0005977	glycogen metabolic process	<5.00E-4
GO:0006144	purine base metabolic process	<5.00E-4
GO:0007006	mitochondrial membrane organization	<5.00E-4
GO:0009156	ribonucleoside monophosphate biosynthetic process	<5.00E-4
GO:0030855	epithelial cell differentiation	<5.00E-4
GO:0046112	nucleobase biosynthetic process	<5.00E-4
GO:0050655	dermatan sulfate proteoglycan metabolic process	<5.00E-4
GO:0009953	dorsal/ventral pattern formation	5.00E-04
GO:0032964	collagen biosynthetic process	5.00E-04
GO:0042273	ribosomal large subunit biogenesis	5.00E-04
GO:0006641	triglyceride metabolic process	0.001
GO:0048469	cell maturation	0.001
GO:0051131	chaperone-mediated protein complex assembly	0.001
GO:0070613	regulation of protein processing	0.001
GO:0007530	sex determination	0.0015
GO:0006368	RNA elongation from RNA polymerase II promoter	0.002
GO:0006414	translational elongation	0.002
GO:0007283	spermatogenesis	0.002
GO:0002698	negative regulation of immune effector process	0.003
GO:0006814	sodium ion transport	0.003
GO:0006813	potassium ion transport	0.004
GO:0007159	leukocyte cell-cell adhesion	0.004
GO:0042446	hormone biosynthetic process	0.004
GO:0050852	T cell receptor signaling pathway	0.0045
GO:0009120	deoxyribonucleoside metabolic process	0.005
GO:0042274	ribosomal small subunit biogenesis	0.005
GO:0006360	transcription from RNA polymerase I promoter	0.0055
GO:0045861	negative regulation of proteolysis	0.006
GO:0051096	positive regulation of helicase activity	0.0065
GO:0006885	regulation of pH	0.0075
GO:0009612	response to mechanical stimulus	0.0075
GO:0034446	substrate adhesion-dependent cell spreading	0.0075
GO:0046320	regulation of fatty acid oxidation	0.0075
GO:0007422	peripheral nervous system development	0.008
GO:0006096	glycolysis	0.0085
GO:0006929	substrate-bound cell migration	0.0085
GO:0060325	face morphogenesis	0.0085
GO:0051668	localization within membrane	0.0095
GO:0030198	extracellular matrix organization	0.01

*Green terms: signal transduction terms; red terms: terms belonging to cell cycle/DNA metabolism –related processes; blue terms: terms belonging to the immune system/stimulus response/cytokine production.

3. MLSA results.

3.1. GSE10255

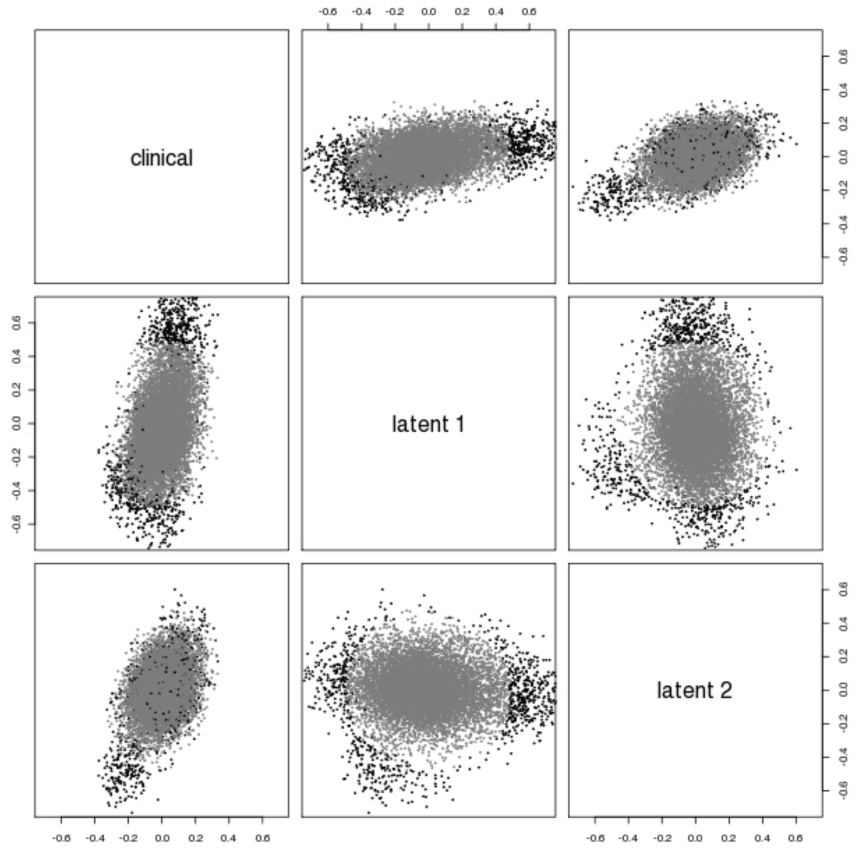

Figure S9. Pair-wise scatterplots of the gene expression projected onto the clinical factor and the two factors found by MLSA. Black points correspond to genes with projection length >0.4 onto the three dimensional subspace..

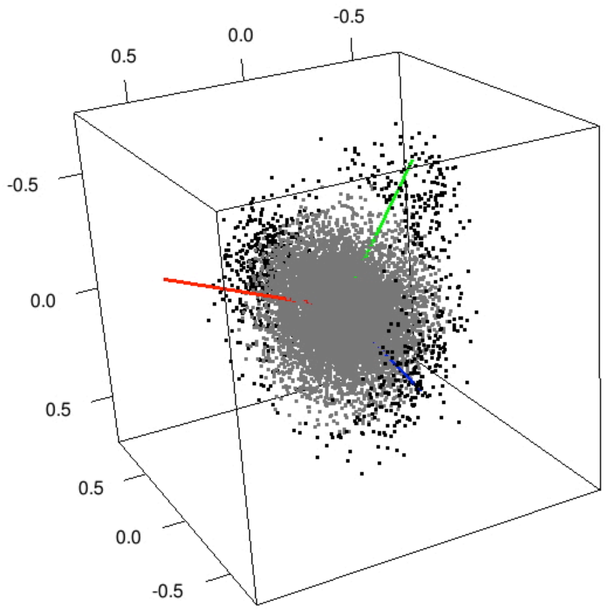

Figure S10. Three-dimensional plot of the genes' projection onto the subspace spanned by the clinical factor and the two factors found by MLSA. The colored axes were results of oblique rotation.

Table S17. Biological processes associated with the rotated axis 1 (p≤0.01).

GOID	Term	pvalue
GO:0002711	positive regulation of T cell mediated immunity	<5.0E-4
GO:0007186	G-protein coupled receptor protein signaling pathway	<5.0E-4
GO:0007268	synaptic transmission	<5.0E-4
GO:0007608	sensory perception of smell	<5.0E-4
GO:0019882	antigen processing and presentation	<5.0E-4
GO:0043123	positive regulation of I-kappaB kinase/NF-kappaB cascade	<5.0E-4
GO:0043368	positive T cell selection	<5.0E-4
GO:0050852	T cell receptor signaling pathway	<5.0E-4
GO:0050853	B cell receptor signaling pathway	<5.0E-4
GO:0050857	positive regulation of antigen receptor-mediated signaling pathway	<5.0E-4
GO:0007156	homophilic cell adhesion	5.00E-04
GO:0008299	isoprenoid biosynthetic process	5.00E-04
GO:0032109	positive regulation of response to nutrient levels	5.00E-04
GO:0043243	positive regulation of protein complex disassembly	5.00E-04
GO:0007283	spermatogenesis	0.001
GO:0050655	dermatan sulfate proteoglycan metabolic process	0.001
GO:0045730	respiratory burst	0.0015
GO:0006783	heme biosynthetic process	0.002
GO:0006936	muscle contraction	0.002
GO:0007160	cell-matrix adhesion	0.002
GO:0007586	digestion	0.002
GO:0007601	visual perception	0.002
GO:0019221	cytokine-mediated signaling pathway	0.002
GO:0045582	positive regulation of T cell differentiation	0.0025
GO:0043010	camera-type eye development	0.003
GO:0050909	sensory perception of taste	0.003
GO:0030574	collagen catabolic process	0.004
GO:0070206	protein trimerization	0.0045
GO:0032364	oxygen homeostasis	0.0055
GO:0006096	glycolysis	0.006
GO:0006916	anti-apoptosis	0.006
GO:0019722	calcium-mediated signaling	0.0065
GO:0007611	learning or memory	0.007
GO:0042116	macrophage activation	0.007
GO:0006839	mitochondrial transport	0.0085
GO:0030183	B cell differentiation	0.0085
GO:0046173	polyol biosynthetic process	0.0085
GO:0010575	positive regulation vascular endothelial growth factor production	0.009
GO:0006584	catecholamine metabolic process	0.0095
GO:0007631	feeding behavior	0.0095
GO:0030198	extracellular matrix organization	0.0095
GO:0045176	apical protein localization	0.0095
GO:0048873	homeostasis of number of cells within a tissue	0.0095

GO:0000122	negative regulation of transcription from RNA polymerase II promoter	0.01
GO:0006469	negative regulation of protein kinase activity	0.01

*Red terms: terms belonging to cell cycle/DNA metabolism – related processes; blue terms: terms belonging to the immune system; purple terms: RNA metabolism/transport terms.

Table S18. Biological processes associated with the rotated axis 2 (p≤0.01).

GOID	Term	pvalue
GO:0000077	DNA damage checkpoint	<5.0E-04
GO:0000079	regulation of cyclin-dependent protein kinase activity	<5.0E-04
GO:0000085	G2 phase of mitotic cell cycle	<5.0E-04
GO:0000086	G2/M transition of mitotic cell cycle	<5.0E-04
GO:0006260	DNA replication	<5.0E-04
GO:0006266	DNA ligation	<5.0E-04
GO:0006281	DNA repair	<5.0E-04
GO:0006310	DNA recombination	<5.0E-04
GO:0006936	muscle contraction	<5.0E-04
GO:0007018	microtubule-based movement	<5.0E-04
GO:0007051	spindle organization	<5.0E-04
GO:0007059	chromosome segregation	<5.0E-04
GO:0007067	mitosis	<5.0E-04
GO:0007126	meiosis	<5.0E-04
GO:0007186	G-protein coupled receptor protein signaling pathway	<5.0E-04
GO:0009263	deoxyribonucleotide biosynthetic process	<5.0E-04
GO:0010212	response to ionizing radiation	<5.0E-04
GO:0030183	B cell differentiation	<5.0E-04
GO:0030261	chromosome condensation	<5.0E-04
GO:0034508	centromere complex assembly	<5.0E-04
GO:0048015	phosphoinositide-mediated signaling	<5.0E-04
GO:0051293	establishment of spindle localization	<5.0E-04
GO:0051299	centrosome separation	<5.0E-04
GO:0051301	cell division	<5.0E-04
GO:0051303	establishment of chromosome localization	<5.0E-04
GO:0051323	metaphase	<5.0E-04
GO:0051436	negative regulation of ubiquitin-protein ligase activity involved in mitotic cell cycle	<5.0E-04
GO:0051437	positive regulation of ubiquitin-protein ligase activity involved in mitotic cell cycle	<5.0E-04
GO:0001822	kidney development	5.00E-04
GO:0006334	nucleosome assembly	5.00E-04
GO:0006414	translational elongation	5.00E-04
GO:0007608	sensory perception of smell	5.00E-04
GO:0008544	epidermis development	5.00E-04
GO:0009952	anterior/posterior pattern formation	5.00E-04
GO:0048704	embryonic skeletal system morphogenesis	5.00E-04
GO:0050853	B cell receptor signaling pathway	5.00E-04
GO:0051647	nucleus localization	5.00E-04

GO:0007605	*sensory perception of sound*	*0.001*
GO:0007586	*digestion*	*0.0015*
GO:0007601	*visual perception*	*0.0015*
GO:0030198	*extracellular matrix organization*	*0.0015*
GO:0043123	*positive regulation of I-kappaB kinase/NF-kappaB cascade*	*0.0015*
GO:0044419	*interspecies interaction between organisms*	*0.0015*
GO:0006144	*purine base metabolic process*	*0.002*
GO:0006821	*chloride transport*	*0.002*
GO:0007160	*cell-matrix adhesion*	*0.002*
GO:0009219	*pyrimidine deoxyribonucleotide metabolic process*	*0.002*
GO:0031503	*protein complex localization*	*0.002*
GO:0016568	*chromatin modification*	*0.0025*
GO:0030326	*embryonic limb morphogenesis*	*0.0025*
GO:0043010	*camera-type eye development*	*0.0025*
GO:0006836	*neurotransmitter transport*	*0.0035*
GO:0045785	*positive regulation of cell adhesion*	*0.0035*
GO:0045786	*negative regulation of cell cycle*	*0.004*
GO:0007179	*transforming growth factor beta receptor signaling pathway*	*0.0045*
GO:0007268	*synaptic transmission*	*0.0045*
GO:0007596	*blood coagulation*	*0.0045*
GO:0018149	*peptide cross-linking*	*0.0045*
GO:0007507	*heart development*	*0.005*
GO:0008038	*neuron recognition*	*0.005*
GO:0009791	*post-embryonic development*	*0.006*
GO:0000080	*G1 phase of mitotic cell cycle*	*0.0065*
GO:0042472	*inner ear morphogenesis*	*0.0065*
GO:0009968	*negative regulation of signal transduction*	*0.0075*
GO:0070206	*protein trimerization*	*0.0075*
GO:0006516	*glycoprotein catabolic process*	*0.008*
GO:0000578	*embryonic axis specification*	*0.0085*
GO:0008217	*regulation of blood pressure*	*0.0085*
GO:0009132	*nucleoside diphosphate metabolic process*	*0.0085*
GO:0046605	*regulation of centrosome cycle*	*0.0085*
GO:0006814	*sodium ion transport*	*0.009*
GO:0019882	*antigen processing and presentation*	*0.009*
GO:0006997	*nucleus organization*	*0.0095*

*Red terms: terms belonging to cell cycle/DNA metabolism – related processes; blue terms: terms belonging to the immune system; purple terms: RNA metabolism/transport terms.

3.2. GSE18864

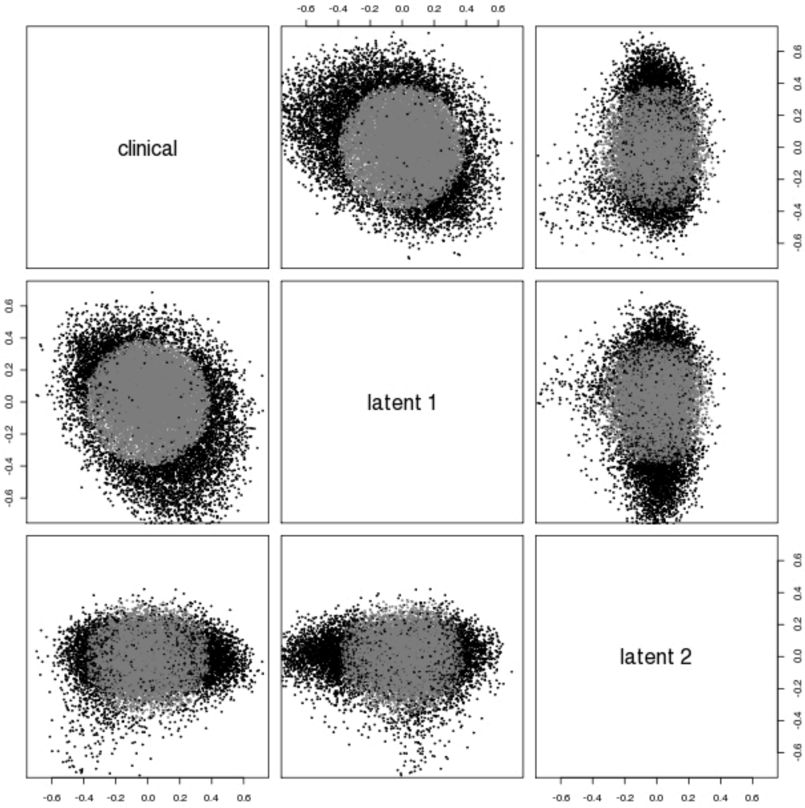

Figure S11. Pair-wise scatterplots of the gene expression projected onto the clinical factor and the two factors found by MLSA. Black points correspond to genes with projection length >0.4 onto the three dimensional subspace.

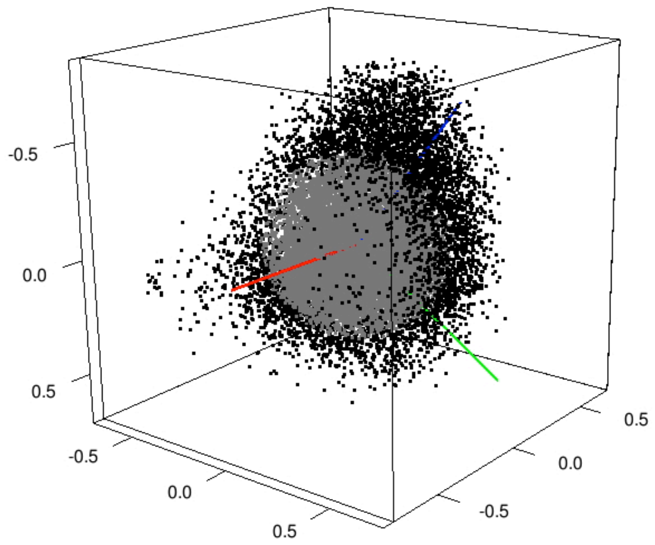

Figure S12. Three-dimensional plot of the genes' projection onto the subspace spanned by the clinical factor and the two factors found by MLSA. The colored axes were results of oblique rotation. The axes were rotated using PCA based only on the points that have projection length of >0.4.

Table S19. Biological processes associated with the rotated axis 1 ($p \le 0.01$).

GOID	*Term*	*pvalue*
GO:0005977	*glycogen metabolic process*	*<5.0E-04*
GO:0006144	*purine base metabolic process*	*<5.0E-04*
GO:0006260	*DNA replication*	*<5.0E-04*

GO:0006281	DNA repair	<5.0E-04
GO:0006401	RNA catabolic process	<5.0E-04
GO:0007051	spindle organization	<5.0E-04
GO:0007059	chromosome segregation	<5.0E-04
GO:0007067	mitosis	<5.0E-04
GO:0030261	chromosome condensation	<5.0E-04
GO:0034508	centromere complex assembly	<5.0E-04
GO:0051096	positive regulation of helicase activity	<5.0E-04
GO:0051299	centrosome separation	<5.0E-04
GO:0051301	cell division	<5.0E-04
GO:0051303	establishment of chromosome localization	<5.0E-04
GO:0000085	G2 phase of mitotic cell cycle	5.00E-04
GO:0007018	microtubule-based movement	5.00E-04
GO:0008380	RNA splicing	5.00E-04
GO:0009156	ribonucleoside monophosphate biosynthetic process	5.00E-04
GO:0010149	senescence	5.00E-04
GO:0045087	innate immune response	5.00E-04
GO:0006397	mRNA processing	0.001
GO:0006874	cellular calcium ion homeostasis	0.001
GO:0006954	inflammatory response	0.0015
GO:0007186	G-protein coupled receptor protein signaling pathway	0.0015
GO:0050909	sensory perception of taste	0.0015
GO:0008217	regulation of blood pressure	0.002
GO:0043094	cellular metabolic compound salvage	0.002
GO:0051028	mRNA transport	0.002
GO:0051293	establishment of spindle localization	0.002
GO:0046112	nucleobase biosynthetic process	0.003
GO:0051323	metaphase	0.003
GO:0006730	one-carbon metabolic process	0.004
GO:0006821	chloride transport	0.004
GO:0007126	meiosis	0.004
GO:0001895	retina homeostasis	0.0045
GO:0045076	regulation of interleukin-2 biosynthetic process	0.0045
GO:0006108	malate metabolic process	0.005
GO:0042036	negative regulation of cytokine biosynthetic process	0.005
GO:0070613	regulation of protein processing	0.005
GO:0006310	DNA recombination	0.0055
GO:0009067	aspartate family amino acid biosynthetic process	0.0055
GO:0009220	pyrimidine ribonucleotide biosynthetic process	0.0055
GO:0007190	activation of adenylate cyclase activity	0.006
GO:0007157	heterophilic cell-cell adhesion	0.0065
GO:0007411	axon guidance	0.007
GO:0046605	regulation of centrosome cycle	0.0075
GO:0006266	DNA ligation	0.0085
GO:0007631	feeding behavior	0.0085

GO:0000082	*G1/S transition of mitotic cell cycle*	*0.009*
GO:0006891	*intra-Golgi vesicle-mediated transport*	*0.0095*
GO:0006892	*post-Golgi vesicle-mediated transport*	*0.01*

*Green terms: signal transduction terms; red terms: terms belonging to cell cycle/DNA metabolism – related processes; blue terms: terms belonging to the immune system/stimulus response/cytokine production.

Table S20. Biological processes associated with the rotated axis 2 (p≤0.01).

GOID	*Term*	*pvalue*
GO:0045861	*negative regulation of proteolysis*	*<5.0E-04*
GO:0007159	*leukocyte cell-cell adhesion*	*5.00E-04*
GO:0007268	*synaptic transmission*	*5.00E-04*
GO:0030036	*actin cytoskeleton organization*	*5.00E-04*
GO:0032964	*collagen biosynthetic process*	*5.00E-04*
GO:0006805	*xenobiotic metabolic process*	*0.001*
GO:0007283	*spermatogenesis*	*0.001*
GO:0006814	*sodium ion transport*	*0.002*
GO:0007586	*digestion*	*0.002*
GO:0007588	*excretion*	*0.002*
GO:0050852	*T cell receptor signaling pathway*	*0.002*
GO:0007156	*homophilic cell adhesion*	*0.0025*
GO:0045123	*cellular extravasation*	*0.003*
GO:0042446	*hormone biosynthetic process*	*0.0035*
GO:0042273	*ribosomal large subunit biogenesis*	*0.004*
GO:0044419	*interspecies interaction between organisms*	*0.004*
GO:0051055	*negative regulation of lipid biosynthetic process*	*0.004*
GO:0007420	*brain development*	*0.005*
GO:0015858	*nucleoside transport*	*0.0055*
GO:0051096	*positive regulation of helicase activity*	*0.0055*
GO:0007589	*body fluid secretion*	*0.006*
GO:0006914	*autophagy*	*0.0065*
GO:0007006	*mitochondrial membrane organization*	*0.0065*
GO:0007601	*visual perception*	*0.0065*
GO:0009953	*dorsal/ventral pattern formation*	*0.0065*
GO:0007416	*synapse assembly*	*0.0075*
GO:0006368	*RNA elongation from RNA polymerase II promoter*	*0.008*
GO:0031663	*lipopolysaccharide-mediated signaling pathway*	*0.0085*
GO:0007190	*activation of adenylate cyclase activity*	*0.0095*
GO:0007224	*smoothened signaling pathway*	*0.0095*
GO:0060558	*regulation of calcidiol 1-monooxygenase activity*	*0.01*

*Green terms: signal transduction terms; red terms: terms belonging to cell cycle/DNA metabolism – related processes; blue terms: terms belonging to the immune system/stimulus response/cytokine production.
